# Dynamic immune changes after bone marrow sparing VMAT in women with locally advanced cervical cancer treated with chemoradiotherapy

**DOI:** 10.1016/j.ctro.2026.101107

**Published:** 2026-01-16

**Authors:** Anouk Corbeau, Marij J.P. Welters, Sanne Boekestijn, Jan Willem M. Mens, Henrike Westerveld, Mila Donker, Laura A. Velema, Hélène van Meir, Mariëtte I.E. van Poelgeest, Judith R. Kroep, Ingrid A. Boere, Sander C. Kuipers, Jeremy Godart, Mischa S. Hoogeman, Hein Putter, Carien L. Creutzberg, Remi A. Nout, Sjoerd H. van der Burg, Stephanie M. de Boer

**Affiliations:** a Department of Radiation Oncology, Leiden University Medical Center, Albinusdreef 2, 2333 ZA Leiden, The Netherlands; b Department of Medical Oncology, Leiden University Medical Center, Leiden, The Netherlands; c Department of Radiotherapy, Erasmus MC Cancer Institute, University Medical Center Rotterdam, Rotterdam, The Netherlands; d Department of Gynecology, Tergooi Medical Center, Hilversum, The Netherlands; e Department of Gynecology, Leiden University Medical Center, Leiden, The Netherlands; f Department of Medical Oncology, Erasmus MC Cancer Institute, University Medical Center Rotterdam, Rotterdam, The Netherlands; g Department of Medical Statistics, Leiden University Medical Center, Leiden, The Netherlands

## Abstract

**Background:**

Chemoradiotherapy is immunosuppressive in women with locally advanced cervical cancer (LACC). Both advanced external-beam radiation therapy (EBRT) and bone marrow sparing (BMS) radiotherapy techniques might lower bone marrow dose and therefore reduce the impact on the immune system. In this study, immune composition and function changes in the blood of women with LACC were evaluated for BMS volumetric-modulated arc therapy (VMAT) and exploratively compared with a historical cohort of women with LACC who underwent non-BMS radiotherapy (RT) with older EBRT techniques.

**Methods:**

Women were treated with chemoradiotherapy followed by brachytherapy according to EMBRACE-II protocol (BMS VMAT) or with 46–52.5 Gy in 23–30 fractions (non-BMS RT). Blood samples for immunomonitoring were collected at set timepoints. Statistical analyses were performed using linear mixed-effects models.

**Results:**

Eighteen and eleven women received BMS VMAT and non-BMS RT, respectively. Although BMS VMAT reduced mean pelvic bone dose by 8.1 Gy, it did not prevent treatment-induced leukopenia and lymphopenia. Chemoradiotherapy mainly reduced CD4+ T helper cells and B cells, leaving CD8+ T-cell and natural killer-cell frequencies untouched. T-cell reactivity to common pathogens was decreased, despite sustained T-cell proliferative capacity, and coincided with increased numbers of regulatory T cells. The potential to activate immune cells remained intact, with small increases in dendritic cells, decreases in myeloid-derived suppressor cells, and preserved capacity of myeloid cells to present antigen and activate T cells.

**Conclusion:**

Our study provided insight in the dynamic immune changes following chemoradiotherapy in LACC. As immunosuppression occurred with BMS VMAT, optimizing BMS and exploring other techniques is warranted.

## Highlights

• Chemoradiotherapy caused immunosuppression enduring 12 months post-treatment.
• Despite bone marrow sparing (BMS) VMAT, all patients developed grade ≥2 lymphopenia.
• T-cell response declined despite better proliferation and stable antigen presentation.
• Cell lineages shifted from lymphoid to myeloid, mainly from CD4+ T helper and B-cell loss.
• Optimizing BMS and exploring other techniques to reduce immunosuppression is needed.

## Introduction

The standard treatment for patients with locally advanced cervical cancer (LACC) combines primary chemoradiotherapy followed by image-guided brachytherapy [1]. However, (chemo)radiotherapy leads to changes in immune cell composition, alters their function, and induces immunosuppression, persisting up to six months after treatment [2], [3]. Hematologic toxicity, especially lymphopenia, reflects the immunosuppressive effect of (chemo)radiotherapy and is commonly observed in patients with LACC [4]. Studies have suggested that radiation-induced lymphopenia is associated with reduced survival in patients receiving radiotherapy for solid tumors [5], [6], [7]. Additionally, combining chemoradiotherapy with adjuvant immunotherapy as a new standard of care for high-risk LACC is gaining interest to improve survival [8]. However, immunotherapy with checkpoint inhibition was less effective in patients with other cancers displaying lymphopenia [9], [10]. The negative impact of lymphopenia on immunotherapy underlines the importance of limiting the immunosuppressive effects of chemoradiotherapy by optimizing radiotherapy techniques, guided by the impact on the immune system.

Bone marrow and integral body dose are associated with immune suppression [11], [12], [13]. The bone marrow serves as a principal home for hematopoietic stem cells, which control the immune cell lineages [14], and integral body dose and/or radiotherapy field size might be a surrogate for dose to the circulating blood, consisting of highly radiosensitive lymphocytes [13], [15], [16]. Reducing dose to both the bone marrow and overall to the body is therefore important and can be achieved using dose-sparing techniques. Firstly, previous studies demonstrated that the introduction of bone marrow sparing (BMS) during radiotherapy planning resulted in a decreased bone marrow dose and reduced radiation-induced lymphopenia in women with LACC [17], [18]. Secondly, advanced external beam radiation (EBRT) techniques, such as intensity-modulated radiotherapy (IMRT) and volumetric-modulated arc therapy (VMAT), provided lower integral body doses [19] and reduced bone marrow doses [20], [21], [22], [23], [24] compared to historical three-dimensional conformal radiotherapy (3DCRT) in gynecological cancers. In contrast, these advanced techniques did not consistently result in a significant decrease in hematologic adverse events [20], [21], [22], [24], [25]. Uncertainties persist regarding the impact of BMS and newer EBRT techniques on hematologic toxicity, and their influence on the composition and function of immune cells are still unexplored.

In view of the ongoing efforts to test adjuvant immunotherapy, this study evaluated the impact of primary chemoradiotherapy using BMS VMAT on the immune system of women with LACC. Changes in T-cell reactivity, antigen-presenting cell capacity, and immune cell composition were investigated up to twelve months after treatment. Additionally, an exploratory comparison was made with a selected patient group from a historical cohort treated with non-BMS 3DCRT/IMRT [3].

## Methods and materials

### Study design and patients

Women with primary LACC treated with primary chemoradiotherapy using BMS VMAT were recruited between May 2022 and January 2024 in the prospective, multicenter, nonrandomized phase-II PROTECT study (NCT05406856) [26]. The study was approved by the Medical Ethics Review Committee Leiden The Hague Delft and Medical Ethics Review Committee Erasmus Medical Center. All participants provided written informed consent.

### Treatment

Patients received BMS VMAT with a total dose of 45 Gy in 25 daily fractions in five weeks according to the EMBRACE-II protocol, using a library-of-plans technique [1]. Based on full and empty bladder planning CT scans, one to three treatment plans were generated to account for uterus motion. Each treatment day the appropriate plan was selected using cone beam-CT. A planning target volume margin (PTV) of 5 mm was used. The whole pelvic bones were delineated on the planning CT scans, extending from 24-25 mm above the upper level of the PTV, including the vertebral column exposed to radiation, down to the inferior border of the ischial tuberosities [27]. Soft dose constraints concerning bone marrow sparing were whole pelvic bones V_10Gy_ < 90 %, V_20Gy_ < 65 %, and V_40Gy_ < 15–20 % [11]. Involved nodes were boosted using a simultaneous integrated boost (SIB) to reach a total equivalent dose of 60 Gy EQD2_10_. Concurrent chemotherapy consisted of weekly cisplatin (40 mg/m^2^) for five weeks. Upon completion of EBRT, MR-guided adaptive brachytherapy was performed with a high-dose rate (HDR) afterloading system aiming for a total equivalent dose (EQD2_10_ D90) between 90 and 95 Gy to at least 90 % of the high-risk clinical target volume (CTV-T_HR_) [1].

### Blood sampling and immunomonitoring analysis

Venous blood was collected before start of radiotherapy (baseline), before the fourth chemotherapy cycle, and at 1, 2, 3, and 12 months after completion of chemoradiotherapy. Blood samples were analyzed for complete blood count, as part of the clinical routine, to determine lymphocyte, leukocyte, neutrophil, and monocyte numbers. Hematologic toxicity was scored according to CTCAE-version 5, and monocytes < 0.10x10^9^/L were considered monocytopenia [28]. Peripheral blood mononuclear cells (PBMCs) were isolated from 45 mL of blood collected in BD vacutainer sodium heparin tubes and used partly freshly, while the remaining cells were cryopreserved for future assays. Similar immunological assays were performed using methodologies previously reported [3]. Briefly, response of T cells to antigens was tested by stimulating freshly obtained PBMCs with influenza matrix 1 overlapping peptides covering the whole protein (FLU) and a memory response mix (MRM) of bacterial recall antigens in a lymphocyte stimulation test (LST). Upon thawing PBMCs, the T-cell proliferative capacity in response to phytohemagglutinin (PHA) was studied as well as the antigen-presenting capacity of these PBMCs in a mixed lymphocyte reaction (MLR). Fold changes in response relative to baseline were calculated. In addition, thawed PBMC samples were subjected to a flow cytometer-based phenotypical identification of immune cell subsets and molecule expression as previously described using a 40-marker panel [29].

### Explorative comparison with historical cohort

The outcomes of this study were exploratively compared with the findings from a historical cohort (2011–2014) [3]. This historical cohort included patients treated with (chemo)radiotherapy for cervical cancer. To enable an appropriate comparison, patients with LACC treated with primary chemoradiotherapy using weekly cisplatin followed by MR-guided adaptive brachytherapy were selected, excluding patients with autoimmune diseases or on immunosuppressive medication. Depending on the year of treatment, patients received non-BMS 3DCRT or IMRT with a dose of 46–48.6 Gy in 23–27 fractions, with one exception where a dose of 52.5 Gy was prescribed [30], [31]. In this treatment period, library-of-plans were not yet used. A PTV margin of 10–15 mm (3DCRT) or 10 mm (IMRT) was used. Sequential boosts (3DCRT) or SIBs (IMRT) were given to involved lymph nodes. Blood samples were taken before start of radiotherapy (baseline), at the 15th EBRT fraction, and 3 weeks after treatment completion and were available for some patients at 6 and 9 weeks after the end of chemoradiotherapy [3].

### Statistical methods

Linear mixed-effects modeling was used to evaluate absolute complete blood counts and fold changes in response relative to baseline for the immunological assays. Fold changes were log10-transformed to reduce skewness. The patient’s identifier was included as random factor and timepoint as fixed factor. For assays with limited blood samples available, timepoints were categorized into baseline, acute (up to 1 month after treatment), and late (more than 1 and up to 12 months after treatment). For the explorative comparison, timepoints were aligned between BMS VMAT and non-BMS 3DCRT/IMRT treatment groups by considering before the fourth chemotherapy cycle, and 1, 2, and 3 months after treatment equivalent to at the 15th EBRT fraction, and 3, 6, and 9 weeks after treatment, respectively. If more than three patients treated with non-BMS 3DCRT/IMRT were available, linear mixed-effects models included both timepoint and treatment as fixed factors. A p-value less than 0.05 was considered statistically significant. Statistical analyses were performed with R (v4.3.1).

## Results

### Patient characteristics

Twenty women were recruited and treated with BMS VMAT, 2 of whom withdrew informed consent before the start of treatment, leaving 18 women for evaluation. Two patients had progressive disease, one immediately after treatment and one at three months, and no further blood samples were collected from these patients. No other patients experienced a relapse during one year of follow-up. Patient characteristics are summarized in Table 1. One patient developed a clinically relevant infection during treatment (pyelonephritis due to nephrostomy drains). The LST could not be performed in all blood samples at all timepoints, as blood volume was limited, and the PHA-proliferation test and MLR could not be performed in 9 of the 18 women due to technical problems during the thawing of cryopreserved PBMCs. The number of blood samples available for each analysis and the median time between end of treatment and the blood sampling are provided in Table S1.

**Table 1:** Patient characteristics of women with locally advanced cervical cancer (LACC) (n = 18) treated with bone marrow sparing (BMS) volumetric-modulated arc therapy (VMAT). *EBRT = external beam radiation therapy, FIGO = International Federation of Gynecology and Obstetrics, PAO = para-aortic, PTV = planning target volume, sd = standard deviation.*

Characteristic	median/mean (range) or n [%]
Median age [years] (range)	52.5 (25 – 77)
Smoking	
yes	2 [11.1 %]
former	4 [22.2 %]
no	12 [66.7 %]
FIGO stage (2018)	
IIB	5 [27.8 %]
IIIB	1 [5.6 %]
IIIC1	12 [66.7 %]
No. of chemotherapy cycles	
1	1 [5.6 %]
2	1 [5.6 %]
4	5 [27.8 %]
5	11 [61.1 %]
Pretreatment lymphocyte count	
median [10^9^/L] (range)	1.85 (0.89 – 2.79)
missing	2 [11.1 %]
Pretreatment neutrophil/lymphocyte ratio	
median (range)	3.26 (1.35 – 8.16)
missing	2 [11.1 %]
PAO lymph node irradiation (according to EMBRACE-II)	
yes	10 [55.6 %]
no	8 [44.4 %]
Mean PTV volume [cm^3^] (sd)	1,445.9 (259.6)
Mean body V_10Gy_ [cm^3^] (sd)	14,286 (3,627.4)
Mean body V_36Gy_ [cm^3^] (sd)	2,304.8 (525.9)
Mean body V_43Gy_ [cm^3^] (sd)	1,494.0 (307.6)
Mean body V_50Gy_ [cm^3^] (sd)	60.5 (59.6)
Mean pelvic bones D_mean_ [Gy] (sd)	24.7 (1.7)
Mean volume pelvic bones [cm^3^] (sd)	1,433.3 (174.1)
Mean V_10Gy_ pelvic bones [%] (sd)	86.1 (3.8)
Mean V_20Gy_ pelvic bones [%] (sd)	63.1 (4.3)
Mean V_40Gy_ pelvic bones [%] (sd)	13.3 (2.9)

### Circulating immune cells

**Fig. 1:**
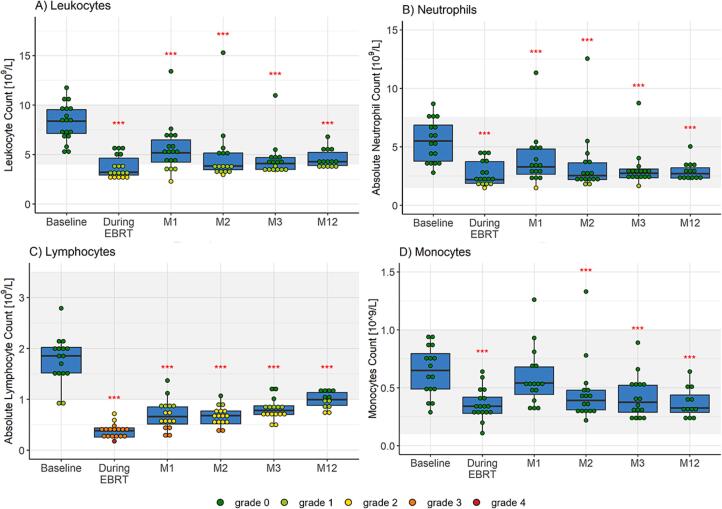
Complete blood cell counts of women with locally advanced cervical cancer (LACC) treated with bone marrow sparing volumetric modulated arc therapy (VMAT). Median fold changes relative to baseline are shown per timepoint. Boxes represent the interquartile range, whiskers indicate the full range, the grey area highlights the normal range according to the clinical practice, and significance is indicated as  = p < 0.001,  = p < 0.01,  = p < 0.05 (linear mixed-effects model). *EBRT = external beam radiation therapy, M = month.*

The number of circulating leukocytes, neutrophils, lymphocytes, and monocytes declined significantly during chemoradiotherapy (all p ≤ 0.001, see Fig. 1, Table S2) and remained at a reduced level up to the twelve-month follow-up. Grade ≥ 2 leukopenia, neutropenia, and lymphopenia were seen in 5 (27.8 %), 1 (5.9 %), and 18 (100 %) women, respectively. There were no differences in circulating immune cell numbers between patients treated with (n = 10) or without (n = 8) para-aortic lymph node irradiation (Fig. S1).

### T-cell response to antigens and mitogens

**Fig. 2:**
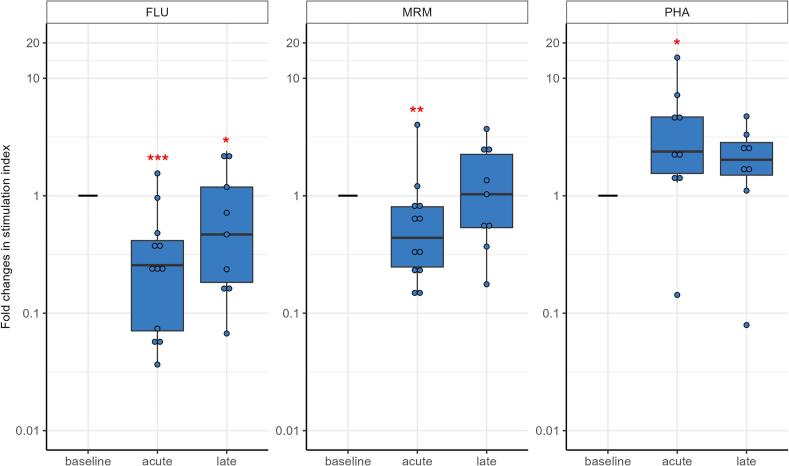
Fold changes in response of T cells to influenza M1 peptides (FLU) and a mix of bacterial recall antigens (MRM), tested with freshly isolated peripheral blood mononuclear cell (PBMCs) in a lymphocyte stimulation test (LST), and to strong mitogen phytohemagglutinin (PHA), using cryopreserved PBMCs in a proliferation test, in women with locally advanced cervical cancer (LACC) treated with bone marrow sparing volumetric-modulated arc therapy (VMAT). Median fold changes relative to baseline are shown for the acute (during treatment and up to 1 month after treatment) and late phase (more than 1 and up to 12 months post-treatment). Boxes represent the interquartile range, whiskers indicate the full range, and significance is indicated as  = p < 0.001,  = p < 0.01,  = p < 0.05 (linear mixed-effects model).

T-cell reactivity to recall antigens of influenza matrix 1 (FLU) declined significantly in the acute phase (up to 1 month after treatment) (p < 0.001) and, although it started to recover, still was lower in the late phase (more than 1 and up to 12 months after treatment) (p = 0.023, Fig. 2, Table S3). Response to a mix of bacterial recall antigens (MRM) declined significantly in the acute phase (p = 0.004), but recovered to baseline level in the late phase. Notably, T cells still displayed the capacity to proliferate to a strong mitogen phytohemagglutinin (PHA), and even was somewhat increased in the acute phase (p = 0.024).

### Antigen-presenting capacity

**Fig. 3:**
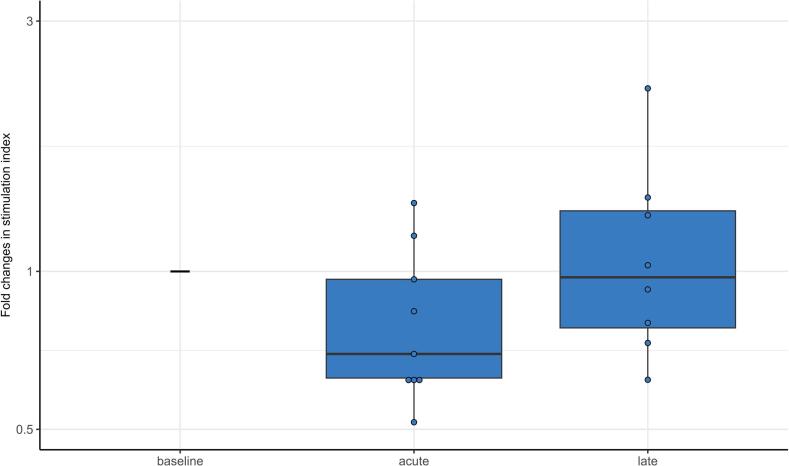
Fold changes in antigen-presenting capacity of peripheral blood mononuclear cell (PBMCs), as determined in a mixed lymphocyte reaction (MLR) in women with locally advanced cervical cancer (LACC) treated with bone marrow sparing volumetric-modulated arc therapy (VMAT). Median fold changes relative to baseline are shown for the acute (during treatment and up to 1 month after treatment) and late phase (more than 1 and up to 12 months post-treatment). Boxes represent the interquartile range, whiskers indicate the full range. None of the fold changes was statistically significant.

The antigen-presenting capacity of myeloid cells within the PBMCs decreased in the acute phase, although not significant (p = 0.095), and was fully recovered to the baseline level in the late phase (Fig. 3, Table S4).

### Immune cell composition

The composition and dynamics in lymphocytes and monocytes from Fig. 1C) and 1D) was further investigated using a 40-color marker panel and spectral flow cytometry-based identification of CD45+ immune cell subsets.

#### Lymphoid cell lineage

**Fig. 4:**
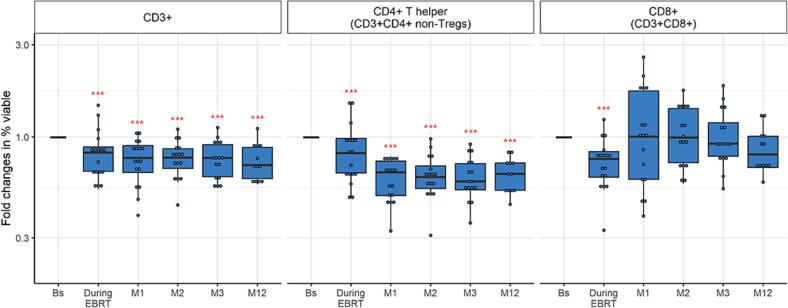
Fold changes in CD3+, CD4+ T helper (CD3+ CD4+ excluding regulatory T cells), and CD8+ (CD3+ CD8+) T-cell frequency, expressed as percentage of all viable cells, as determined with flow cytometry in women with locally advanced cervical cancer (LACC) treated with bone marrow sparing volumetric-modulated arc therapy (VMAT). Median fold changes relative to baseline are shown per timepoint. Boxes represent the interquartile range, whiskers indicate the full range, and significance is indicated as  = p < 0.001,  = p < 0.01,  = p < 0.05 (linear mixed-effects model).

The relative frequency of CD3+, CD4+ T helper cells (CD3+CD4+ excluding regulatory T cells (Tregs)), and CD8+ (CD3+CD8+) T cells, essential for the adaptive immune response, decreased significantly during EBRT compared to pretreatment (p ≤ 0.001) (Fig. 4, Table S5). The frequency of CD3+ and CD4+ T helper cells remained significantly reduced up to twelve months after treatment (p < 0.001), whereas CD8+ T cells recovered to baseline level at one month post-treatment.

The frequency of CD4+ T helper cells expressing the co-inhibitory marker program death-1 (PD-1) was higher at all timepoints compared to baseline (p < 0.001), whereas this remained unchanged for CD8+ T cells (Fig. S2). Other inhibitory and activation markers that showed an increased expression due to chemoradiotherapy were KLRG1, Tim-3, ICOS, and HLA-DR. Immunosuppressive regulatory T cells (Tregs, CD3+CD4+CD25+CD127-Foxp3+) and more precise activated Tregs (CD3+CD4+CD25+ CD127-Foxp3+ CD45RA-) were elevated at all timepoints during and after therapy (p < 0.001).

**Fig. 5:**
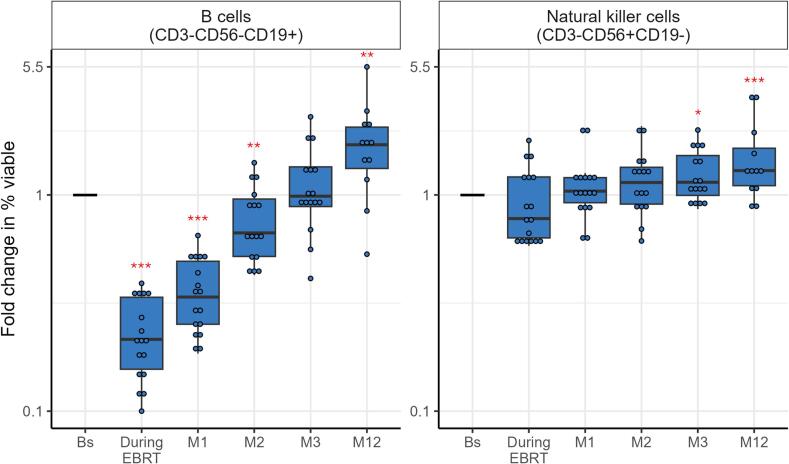
Fold changes over time for expression of B cells and natural killer cells, expressed as percentage of viable cells, in women with locally advanced cervical cancer (LACC) treated with bone marrow sparing volumetric-modulated arc therapy (VMAT). Median fold changes relative to baseline are shown per timepoint. Boxes represent the interquartile range, whiskers indicate the full range, and significance is indicated as  = p < 0.001,  = p < 0.01,  = p < 0.05 (linear mixed-effects model).

B-cell numbers (CD3-CD56-CD19+) declined during EBRT and remained lower up to 2 months post-treatment (p < 0.01), followed by a recovery and even an increase at 12 months post-treatment (p < 0.01) (Fig. 5, Table S6). Natural killer cell (CD3-CD56+CD19-) numbers increased at 3 (p < 0.05) and 12 (p < 0.001) months post-treatment.

#### Myeloid lineage

**Fig. 6:**
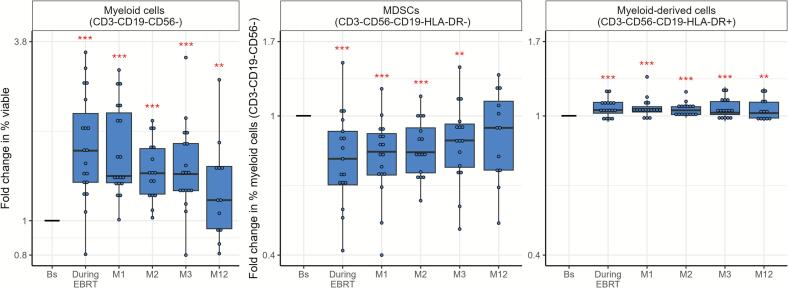
Fold changes over time for expression of myeloid cells (CD3-CD19-CD56-), as percentage of viable cells, and within this group the expression of myeloid-derived suppressor cells (MDSCs) (HLA-DR-) and myeloid-derived cells (HLA-DR+) in women with locally advanced cervical cancer (LACC) treated with bone marrow sparing volumetric-modulated arc therapy (VMAT). Median fold changes relative to baseline are shown per timepoint. Boxes represent the interquartile range, whiskers indicate the full range, and significance is indicated as  = p < 0.001,  = p < 0.01,  = p < 0.05 (linear mixed-effects model).

Myeloid cells (CD3-CD19-CD56-) increased at all timepoints compared to baseline (p < 0.01, Fig. 6, Table S7). Within the myeloid lineage, both groups showed decreased myeloid-derived suppressor cells (MDSCs) (CD3-CD19-CD56-HLA-DR-) and increased myeloid-derived cells (CD3-CD19-CD56-HLA-DR+) up to 12 and 3 months post-treatment, respectively. Among the myeloid-derived cells, two subtypes of macrophage-like cells (CD3-CD19-CD56-HLA-DR+CD11b+CD14+ and CD3-CD19-CD56-HLA-DR+CD11b++CD14++) increased during treatment and subsequently declined, whereas dendritic cells (CD3-CD19-CD56-HLA-DR+CD11b-CD14-CD11c+) had the opposite pattern (Fig. S3).

### Explorative comparison with historical cohort

The historical cohort included 30 women with advanced cervical cancer of whom 15 had received primary chemoradiotherapy using non-BMS 3DCRT/IMRT with brachytherapy [3]. However, 2 participants withdrew after the first blood sample and for 2 participants no EBRT treatment plans were available, leaving 11 available for the explorative comparison. Six patients had given additional consent for blood samples at 6 and 9 weeks after treatment and in three participants response to PHA and flow cytometry measurements was assessed (Table S1). Table S8 provides the patient characteristics.

### Circulating immune cells

**Fig. 7:**
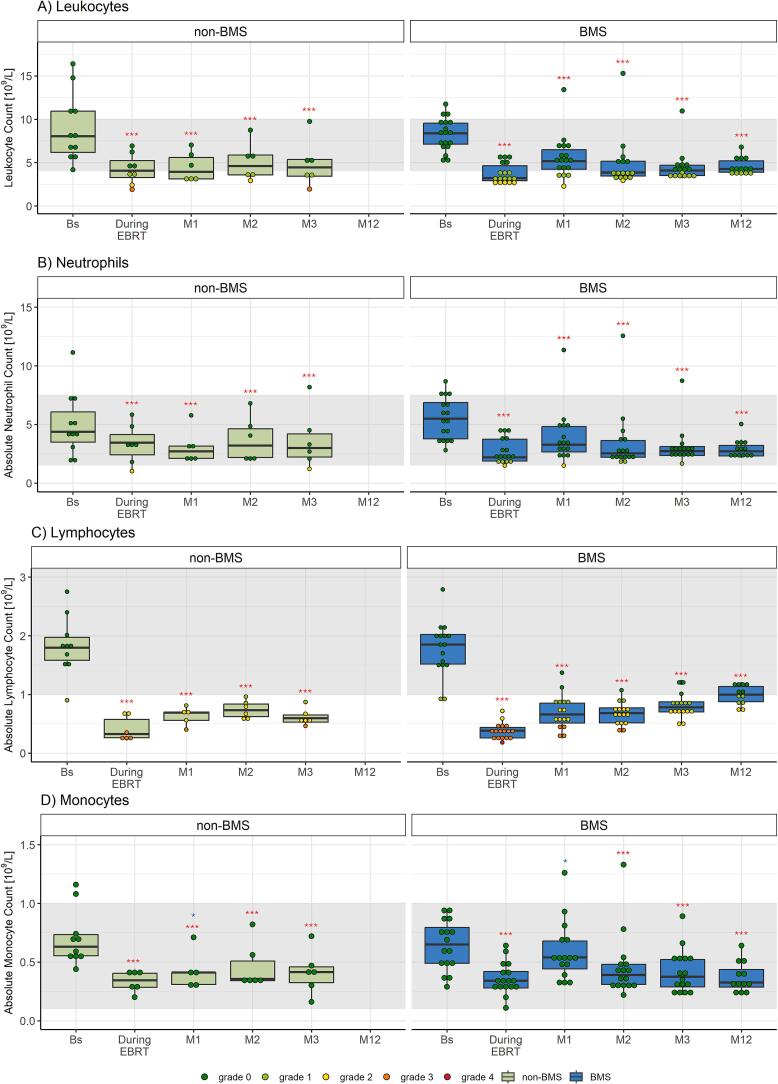
Complete blood cell counts of women with locally advanced cervical cancer (LACC) treated with chemoradiotherapy with three dimensional conformal radiation therapy (3DCRT) or intensity-modulated radiation therapy (IMRT) without bone marrow sparing (BMS) (non-BMS group) or with volumetric-modulated arc therapy (VMAT) with bone marrow sparing (BMS group). Median fold changes relative to baseline are shown per timepoint. Boxes represent the interquartile range, whiskers indicate the full range, the grey area highlights the normal range according to the clinical practice, and significance is indicated as  = p < 0.001,  = p < 0.01,  = p < 0.05, and also compared among the two treatments, with  = p < 0.05 (linear mixed-effects model). *EBRT = external beam radiation therapy, M = month.*

Similar to BMS VMAT, the number of circulating leukocytes, neutrophils, and monocytes was significantly reduced at all timepoints with non-BMS 3DCRT/IMRT (all p < 0.001, Fig. 7, Table S9). The number of circulating immune cells did not differ between the two groups, except for a slightly higher number of monocytes with BMS VMAT at one month post-treatment (p = 0.045).

### T-cell response to antigens and mitogens

**Fig. 8:**
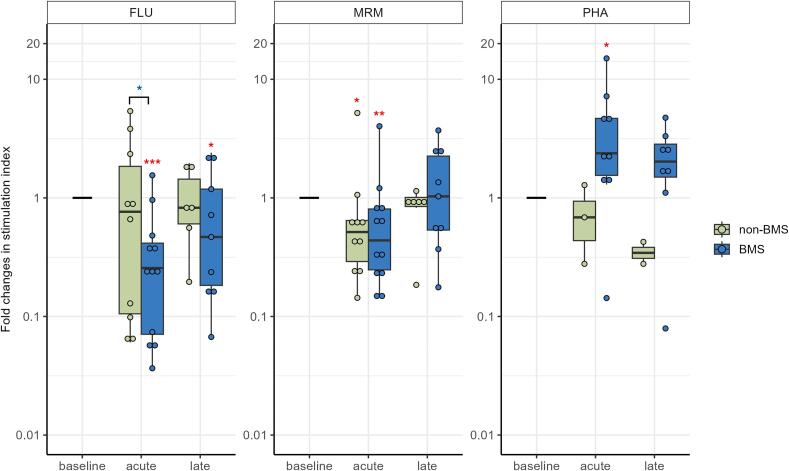
Fold changes in response of T cells to antigens Influenza M1 peptides (FLU) and a mix of bacterial recall antigens (MRM), tested with freshly isolated peripheral blood mononuclear cell (PBMCs) in a lymphocyte stimulation test (LST), and to strong mitogen phytohemagglutinin (PHA), studied using cryopreserved PBMCs in a proliferation test, in women with locally advanced cervical cancer (LACC) treated with chemoradiotherapy with three dimensional conformal radiation therapy (3DCRT) or intensity-modulated radiation therapy (IMRT) without bone marrow sparing (BMS) (non-BMS group) or with volumetric-modulated arc therapy (VMAT) with bone marrow sparing (BMS group). Median fold changes relative to baseline are shown per timepoint. Boxes represent the interquartile range, whiskers indicate the full range, and significance is indicated as  = p < 0.001,  = p < 0.01,  = p < 0.05, and also compared among the two treatments, with  = p < 0.05 (linear mixed-effects model). PHA was not statistically tested in the non-BMS group as there were only three patients available for analysis.

T-cell reactivity to FLU remained unchanged for non-BMS 3DCRT/IMRT and was significantly higher in the acute phase when compared to BMS VMAT (p = 0.045, Fig. 8, Table S10). Response to MRM showed similar patterns for both treatments. T-cell proliferative capacity in response to the PHA was decreased compared to BMS VMAT.

### Antigen-presenting capacity

For non-BMS 3DCRT/IMRT, antigen-presenting capacity was lower in both the acute and late phase compared to BMS VMAT and did not recover to baseline values (Fig. S4).

### Immune cell composition

The relative frequency of CD3+, CD4+ T helper and CD8+ T cells was comparable between the non-BMS 3DCRT/IMRT and BMS VMAT treatment groups (Fig. S5). Similarly, the patterns of inhibitory markers PD-1 and Tim-3, as well as the frequency of the immunosuppressive (a)Tregs, showed no differences between the two treatments (Fig. S6). B-cell and natural killer cell populations remained unchanged in the non-BMS 3DCRT/IMRT group, whereas these changed over time in the BMS VMAT group (Fig. S7). In addition, both non-BMS 3DCRT/IMRT and BMS VMAT showed similar patterns in myeloid cells, MDSCs, and myeloid-derived cells (Fig. S8), as well as in dendritic cells and macrophage-like cells (Fig. S9).

## Discussion

This study showed that chemoradiotherapy in women with locally advanced cervical cancer results in systemic immunosuppression up to twelve months post-treatment. Although bone marrow sparing VMAT resulted in lower pelvic bones dose and reduced volumes of the body receiving 36, 43, and 50 Gy, it did not decrease hematologic toxicity compared to non-bone marrow sparing 3DCRT/IMRT. Despite the somewhat increased potential of circulating T cells to proliferate upon stimulation, the antigen response was reduced, albeit transiently. Potentially, this was due to a chemoradiotherapy-induced decrease in CD4+ T helper cells and B cells, persisting up to twelve months post-treatment and coinciding with an increased frequency of immune-suppressive Tregs. It was less likely the result of changes in the antigen-presenting capacity of the circulating myeloid cells used to test T-cell responsiveness, as the percentage of dendritic cells tended to increase while that of myeloid-derived suppressor cells tended to decrease, fitting with a sustained capacity of myeloid cells to activate T cells. Notably, chemoradiotherapy may also activate populations of T cells and myeloid cells, as reflected by the upregulation of HLA-DR (both T cells and myeloid cells) and other activation and inhibitory molecules (T cells) on circulating immune cells, indicating complex changes in immune regulation.

In our study, bone marrow sparing (BMS) VMAT resulted in absolute reductions of 4.7 %, 20.1 %, and 23.4 % in the volume of the whole pelvic bone receiving a dose of 10, 20, and 40 Gy (V_10Gy_, V_20Gy_, and V_40Gy_), respectively, as compared to non-BMS 3DCRT/IMRT, but did not reduce hematologic toxicity by CTCAE definitions. In contrast, a recent systematic review comparing BMS with non-BMS in cervical cancer reported that pooled reductions of 4.6 %, 10.9 %, and 7.3 %, respectively, reduced both grade ≥ 2 and ≥ 3 hematologic toxicities [17]. On one hand, due to the PROTECT study design, a higher proportion of patients received prophylactic para-aortic irradiation in the BMS VMAT group, even though para-aortic irradiation did not substantially affect the immune parameters in our study. On the other hand, the BMS VMAT group had a lower mean PTV volume and reduced body V_36Gy_-V_50Gy_ due the library-of-plans technique, smaller PTV margins, and more advanced EBRT techniques. Variability in multiple important factors, together with a limited number of patients and blood samples, complicate direct comparisons between BMS and non-BMS and between EBRT techniques.

Previous studies in cervical cancer also showed a reduced number of CD3+ and CD4+ T cells in blood at the end of chemoradiotherapy, but had a limited follow-up time and did not evaluate specifically CD4+ T helper cells [32], [33], [34]. We expanded their findings by showing that this suppressed hematologic immune state persists for at least 12 months post-treatment. Additionally, these studies detected that the CD8+ T cell number declined during or were at the same level after chemoradiotherapy compared to that at baseline [32], [33], [34]. This is supported by our results, as in our study CD8+ T-cell numbers declined during BMS VMAT, but already recovered at one month post-treatment. Two other studies reported a decrease in B and natural killer cells during chemoradiotherapy followed by (almost) recovery at two to three months [35] or six months post-treatment [36], a similar observation was made in our study. Due to deviating analysis of T cells in these latter two studies, we could not compare those outcomes. Thus, our results suggest that circulating CD4+ T cells, and in particular the CD4+ T helper cells, are more affected by chemoradiotherapy than CD8+ T cells, B cells, and natural killer cells.

T cells and myeloid cells showed evidence of activation since expression of HLA-DR and other activation and inhibitory molecules increased due to treatment. Activated T cells may migrate into tissues and therefore disappear from circulating blood [37], [38]. Additionally, the upregulation of immune checkpoint-related molecules such as including PD-1, Tim-3, and ICOS may be linked to responsiveness to immune checkpoint inhibitors, but may also indicate T-cell exhaustion in case of co-expression.

Previous studies evaluating adjuvant immunotherapy in high-risk LACC have reported mixed outcomes [8], [39]. Our study gave insight in the dynamic immune cell composition and function changes, and indicated that specifically the CD4+ T helper cell component was affected. In several studies, pre-treatment levels of circulating CD4+ T helper cells were associated with better response to immunotherapy [40], suggesting that their decrease may negatively impact immunotherapy. The increase in circulating CD4+ Tregs may also contribute to immunotherapy resistance [41]. Thus, further studies are needed to evaluate the relationship between circulating and intratumoral immune cell function and checkpoint molecule expression, after chemoradiotherapy to clarify how these features relate to immunotherapy efficacy.

A previous analysis in the total non-BMS 3DCRT/IMRT cohort did not find a difference in immune cell function and numbers between chemoradiotherapy and radiotherapy alone [3]. Changes in immune cell frequency and function might therefore be mainly radiotherapy-related, highlighting the necessity to optimize the radiotherapy part of the treatment.

Planning studies have shown that intensity-modulated proton therapy (IMPT) can further reduce bone marrow and integral body dose compared to IMRT and VMAT, potentially resulting in decreased hematologic toxicity [42], [43], [44]. Previous studies investigating proton therapy in gynecological cancers were limited by the lack of a comparative arm and included heterogeneous patient groups [45], [46]. Studies, such as the ongoing phase-II PROTECT study, directly comparing bone marrow sparing IMRT/VMAT with bone marrow sparing IMPT in a homogeneous patient cohort will provide more insight into the potential benefits of IMPT in reducing bone marrow toxicity in clinical practice [26].

The main limitation of this study is the limited number of patients and blood samples available in both treatment groups. Additionally, dosimetric differences between the groups could not only be related to BMS and treatment techniques, but also to differences in numbers of patients receiving para-aortic irradiation, the use of library-of-plans, and the smaller margins used. Furthermore, variations in instruments, sample preparation, antibody panels, and technicians between the cohorts may have influenced the immune cell analyses. Moreover, the treatment set up of the patients does not allow us to dissect the individual contributions of chemotherapy and radiotherapy to the observed immunosuppression. Lastly, we analyzed systemic immunity, but further studies should be performed to translate our findings to the tumor microenvironment and to compare treatment effects between circulating and tumor-infiltrating immune cells.

In conclusion, chemoradiotherapy reduced the number of circulating immune cells, specifically CD4+ T helper cells and B cells, as well as lowered antigen-specific T-cell responses, coinciding with increased levels of regulatory T cells in women with locally advanced cervical cancer. In an exploratory comparison with non-bone marrow sparing 3DCRT/IMRT, bone marrow sparing VMAT was associated with improved proliferative capacity of T cells and antigen-presenting capacity with a concomitant decrease in immunosuppressive MDSCs and increase of activated myeloid-derived cells in the blood. Our study provided insight in the dynamic immune cell composition and function changes with bone marrow sparing VMAT and explored the comparison with non-bone marrow sparing 3DCRT/IMRT. Further studies to determine whether similar treatment effects occur within the tumor microenvironment are warranted. As immunosuppression occurred with both treatments, studies optimizing bone marrow sparing or investigating the potential of IMPT in clinical practice, such as the ongoing PROTECT study, are necessary to explore options to reduce bone marrow toxicity in cervical cancer.

## CRediT authorship contribution statement

**Anouk Corbeau:** Conceptualization, Data curation, Formal analysis, Investigation, Methodology, Project administration, Resources, Software, Visualization, Writing – original draft, Writing – review & editing. **Marij J.P. Welters:** Conceptualization, Data curation, Investigation, Methodology, Project administration, Resources, Validation, Writing – original draft, Writing – review & editing. **Sanne Boekestijn:** Data curation, Investigation, Project administration, Resources, Validation, Writing – review & editing. **Jan Willem M. Mens:** Conceptualization, Investigation, Resources, Writing – review & editing. **Henrike Westerveld:** Investigation, Resources, Writing – review & editing. **Mila Donker:** Investigation, Resources, Writing – review & editing. **Laura A. Velema:** Investigation, Resources, Writing – review & editing. **Hélène van Meir:** Data curation, Investigation, Resources, Writing – review & editing. **Mariëtte I.E. van Poelgeest:** Conceptualization, Writing – review & editing. **Judith R. Kroep:** Conceptualization, Writing – review & editing. **Ingrid A. Boere:** Conceptualization, Writing – review & editing. **Sander C. Kuipers:** Conceptualization, Writing – review & editing. **Jeremy Godart:** Conceptualization, Funding acquisition, Investigation, Resources, Writing – review & editing. **Mischa S. Hoogeman:** Conceptualization, Funding acquisition, Writing – review & editing. **Hein Putter:** Methodology, Formal analysis, Writing – review & editing. **Carien L. Creutzberg:** Conceptualization, Funding acquisition, Investigation, Methodology, Resources, Supervision, Writing – review & editing. **Remi A. Nout:** Conceptualization, Funding acquisition, Methodology, Supervision, Writing – review & editing. **Sjoerd H. van der Burg:** Conceptualization, Methodology, Resources, Supervision, Writing – review & editing. **Stephanie M. de Boer:** Conceptualization, Funding acquisition, Methodology, Project administration, Resources, Supervision, Writing – review & editing.

## Declaration of competing interest

The authors declare the following financial interests/personal relationships which may be considered as potential competing interests: Remi Nout, Jeremy Godart, Mischa Hoogeman, Stephanie de Boer, Carien Creutzberg (all paid to institution) report financial support provided by Varian Medical Systems, Inc., a Siemens Healthineers Company. Remi Nout reports a relationship with Elekta, Accuray Inc, Sensius, Senewald that includes: funding grants. Remi Nout reports a relationship with Elekta, MSD that includes: consulting or advisory. Mischa Hoogeman reports a relationship with Accuray Inc, Elekta AB, Varian, Raysearch that includes: funding grants. Mischa Hoogeman reports a test and feedback agreement with Siemens Healthineers (Erlangen, Germany) and participation in a ThinkTank meeting for Accuracy Inc. Carien Creutzberg reports compensation to institution for membership of DMC. If there are other authors, they declare that they have no known competing financial interests or personal relationships that could have appeared to influence the work reported in this paper.
