# Supplementary material for: Dynamic immune changes after bone marrow sparing VMAT in women with locally advanced cervical cancer treated with chemoradiotherapy
Source: Clin Transl Radiat Oncol. 2026 Jan 16;58:101107. doi: 10.1016/j.ctro.2026.101107 (PMC12887253; doi:10.1016/j.ctro.2026.101107)
Supplement: Supplementary Data 1 [file mmc1.docx]

**Dynamic immune changes after bone marrow sparing VMAT in women with locally advanced cervical cancer treated with chemoradiotherapy**

**Supplementary Material**

**Table S1:** Number of blood samples available per timepoint and per immune cell analysis. *BMS = bone marrow sparing, VMAT = volumetric-modulated arc therapy, 3DCRT = three-dimensional conformal radiotherapy, IMRT = intensity-modulated radiotherapy, LST = lymphocyte stimulation test, FLU = influenza matrix 1 overlapping peptides, MRM = memory response mix, PHA = phytohemagglutinin, MLR = mixed lymphocyte reaction, EBRT = external beam radiation therapy, NA = not applicable*

| Analyses | | | Timepoints | Number of samples | |
| --- | --- | --- | --- | --- | --- |
|  |  |  |  | **BMS VMAT**  **(n = 18)** | **non-BMS 3DCRT/IMRT**  **(n = 11)** |
| LST | | **FLU** | Baseline | 17 | 11 |
|  |  |  | During EBRT | 5 | 9 |
|  |  |  | 1 month/3 weeks | 11 | 10 |
|  |  |  | 2 months/6 weeks | 4 | 5 |
|  |  |  | 3 months/9 weeks | 7 | 6 |
|  |  |  | 12 months | 2 | *NA* |
|  |  | **MRM** | Baseline | 17 | 11 |
|  |  |  | During EBRT | 5 | 9 |
|  |  |  | 1 month/3 weeks | 11 | 10 |
|  |  |  | 2 months/6 weeks | 4 | 6 |
|  |  |  | 3 months/9 weeks | 7 | 6 |
|  |  |  | 12 months | 2 | *NA* |
| Proliferation test | | **PHA** | Baseline | 9 | 3 |
|  |  |  | During EBRT | 8 | 2 |
|  |  |  | 1 month/3 weeks | 8 | 2 |
|  |  |  | 2 months/6 weeks | 7 | 0 |
|  |  |  | 3 months/9 weeks | 8 | 2 |
|  |  |  | 12 months | 6 | *NA* |
| MLR | | | Baseline | 9 | 3 |
|  |  |  | During EBRT | 9 | 2 |
|  |  |  | 1 month/3 weeks | 8 | 2 |
|  |  |  | 2 months/6 weeks | 7 | 0 |
|  |  |  | 3 months/9 weeks | 8 | 2 |
|  |  |  | 12 months | 6 | *NA* |
| Blood cell counts | **Leukocytes** | | Baseline | 18 | 11 |
|  |  |  | During EBRT | 18 | 8 |
|  |  |  | 1 month/3 weeks | 18 | 6 |
|  |  |  | 2 months/6 weeks | 16 | 6 |
|  |  |  | 3 months/9 weeks | 16 | 6 |
|  |  |  | 12 months | 14 | *NA* |
|  | **Neutrophils** | | Baseline | 17 | 11 |
|  |  |  | During EBRT | 17 | 7 |
|  |  |  | 1 month/3 weeks | 16 | 6 |
|  |  |  | 2 months/6 weeks | 16 | 6 |
|  |  |  | 3 months/9 weeks | 16 | 6 |
|  |  |  | 12 months | 13 | *NA* |
|  | **Lymphocytes** | | Baseline | 16 | 10 |
|  |  |  | During EBRT | 17 | 6 |
|  |  |  | 1 month/3 weeks | 16 | 5 |
|  |  |  | 2 months/6 weeks | 16 | 6 |
|  |  |  | 3 months/9 weeks | 16 | 6 |
|  |  |  | 12 months | 12 | *NA* |
|  | **Monocytes** | | Baseline | 16 | 10 |
|  |  |  | During EBRT | 17 | 6 |
|  |  |  | 1 month/3 weeks | 16 | 5 |
|  |  |  | 2 months/6 weeks | 16 | 6 |
|  |  |  | 3 months/9 weeks | 16 | 6 |
|  |  |  | 12 months | 12 | *NA* |
| Flow cytometer-based phenotypical identification | | | Baseline | 18 | 3 |
|  |  |  | During EBRT | 17 | 2 |
|  |  |  | 1 month/3 weeks | 17 | 2 |
|  |  |  | 2 months/6 weeks | 16 | 0 |
|  |  |  | 3 months/9 weeks | 16 | 2 |
|  |  |  | 12 months | 12 | *NA* |
| Median time after end of treatment (range) [weeks] | | | 1 month/3 weeks | 4.1 (2.0-5.3) | 4.1 (2.4-5.3) |
|  |  |  | 2 months/6 weeks | 8.6 (7.0-10.4) | 8.5 (7.0-10.6) |
|  |  |  | 3 months/9 weeks | 13.2 (11.4-18.1) | 12.5 (11.0-14.3) |
|  |  |  | 12 months | 56.1 (50.1-62.0) | *NA* |

**Table S2**: Linear mixed-effects models for complete blood cell counts of women with locally advanced cervical cancer (LACC) treated with chemoradiotherapy bone marrow sparing volumetric-modulated arc therapy (VMAT). *EBRT = external beam radiation therapy, M = month*

1. Leukocytes

| Characteristic | Estimate | Standard Error | p-value |
| --- | --- | --- | --- |
| *Intercept* | 8.24 | 0.56 |  |
| *Timepoint* |  |  |  |
| During EBRT | -4.5 | 0.53 | <0.001^*^ |
| 1M after treatment | -2.66 | 0.53 | <0.001^*^ |
| 2M after treatment | -3.41 | 0.55 | <0.001^*^ |
| 3M after treatment | -3.72 | 0.55 | <0.001^*^ |
| 12M after treatment | -3.36 | 0.58 | <0.001^*^ |

*^*^ = statistically significant*

1. Neutrophils

| Characteristic | Estimate | Standard Error | p-value |
| --- | --- | --- | --- |
| *Intercept* | 5.52 | 0.47 |  |
| *Timepoint* |  |  |  |
| During EBRT | -2.81 | 0.47 | <0.001^*^ |
| 1M after treatment | -1.65 | 0.48 | <0.001^*^ |
| 2M after treatment | -2.14 | 0.48 | 0.001^*^ |
| 3M after treatment | -2.40 | 0.48 | <0.001^*^ |
| 12M after treatment | -2.34 | 0.52 | <0.001^*^ |

*^*^ = statistically significant*

1. Lymphocytes

| Characteristic | Estimate | Standard Error | p-value |
| --- | --- | --- | --- |
| *Intercept* | 1.78 | 0.08 |  |
| *Timepoint* |  |  |  |
| During EBRT | -1.39 | 0.07 | <0.001^*^ |
| 1M after treatment | -1.09 | 0.07 | <0.001^*^ |
| 2M after treatment | -1.10 | 0.07 | <0.001^*^ |
| 3M after treatment | -0.98 | 0.07 | <0.001^*^ |
| 12M after treatment | -0.75 | 0.08 | <0.001^*^ |

*^*^ = statistically significant*

1. Monocytes

| Characteristic | Estimate | Standard Error | p-value |
| --- | --- | --- | --- |
| *Intercept* | 0.64 | 0.05 |  |
| *Timepoint* |  |  |  |
| During EBRT | -0.25 | 0.05 | <0.001^*^ |
| 1M after treatment | -0.06 | 0.05 | 0.214 |
| 2M after treatment | -0.18 | 0.05 | <0.001^*^ |
| 3M after treatment | -0.21 | 0.05 | <0.001^*^ |
| 12M after treatment | -0.22 | 0.05 | <0.001^*^ |

*^*^ = statistically significant*

**
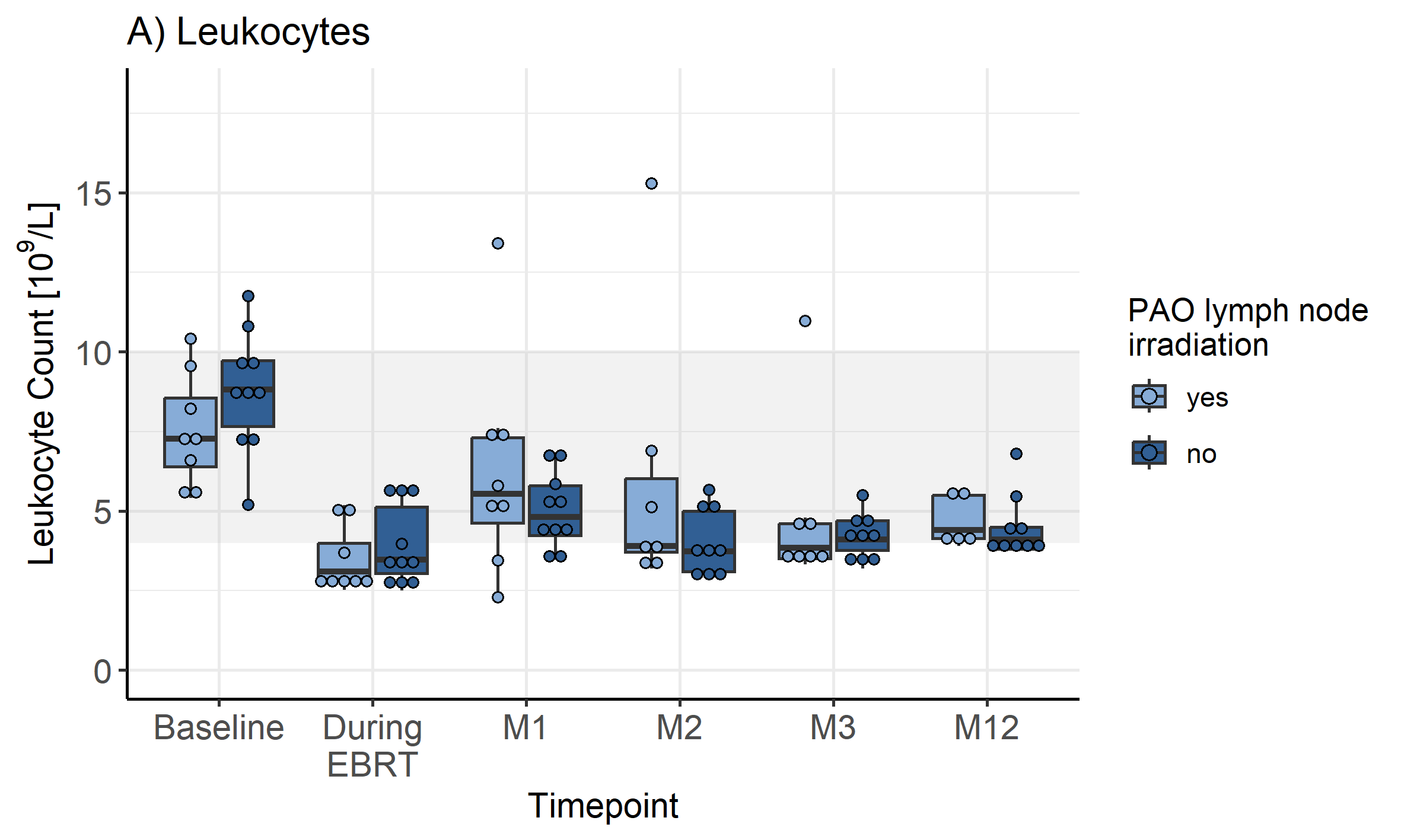

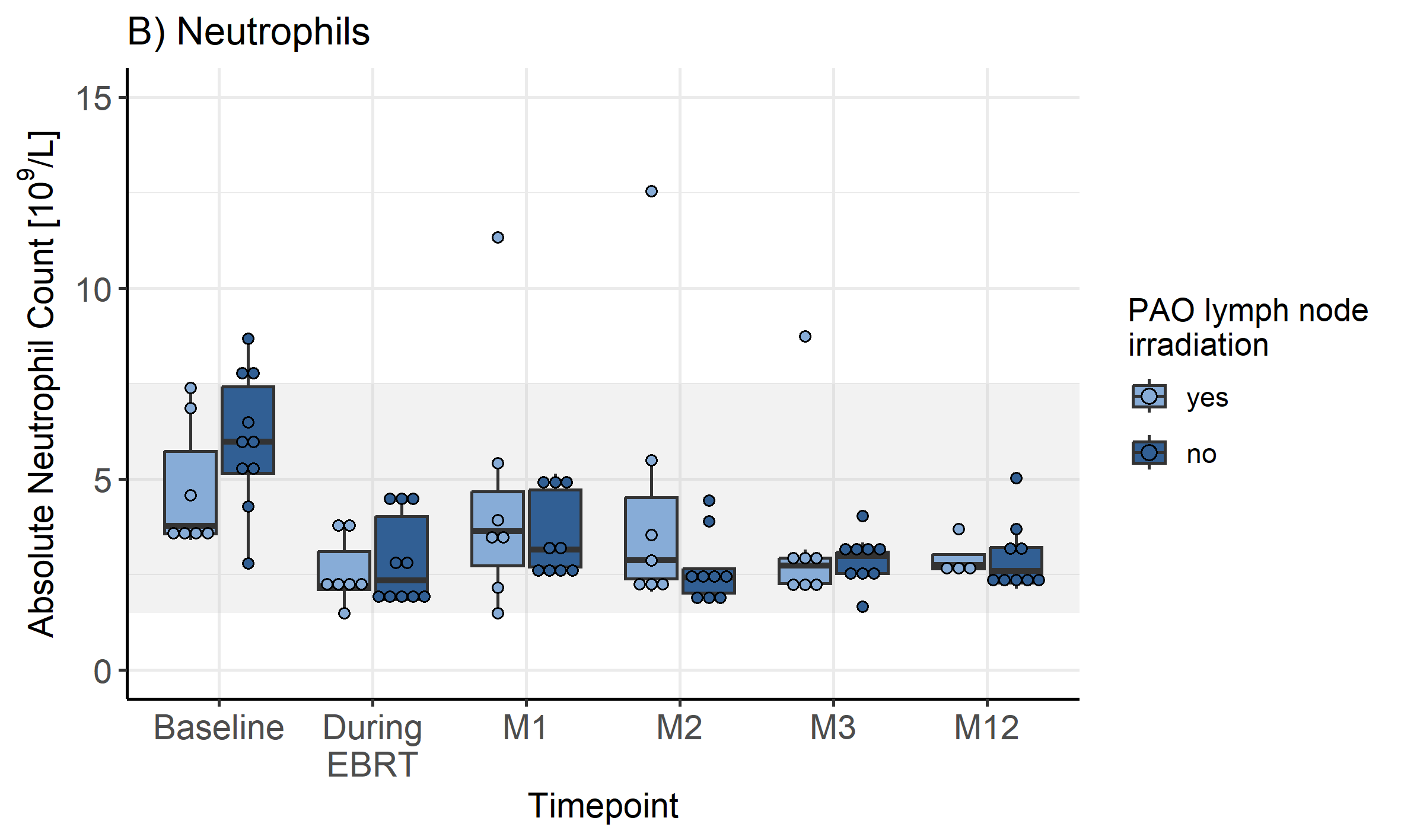

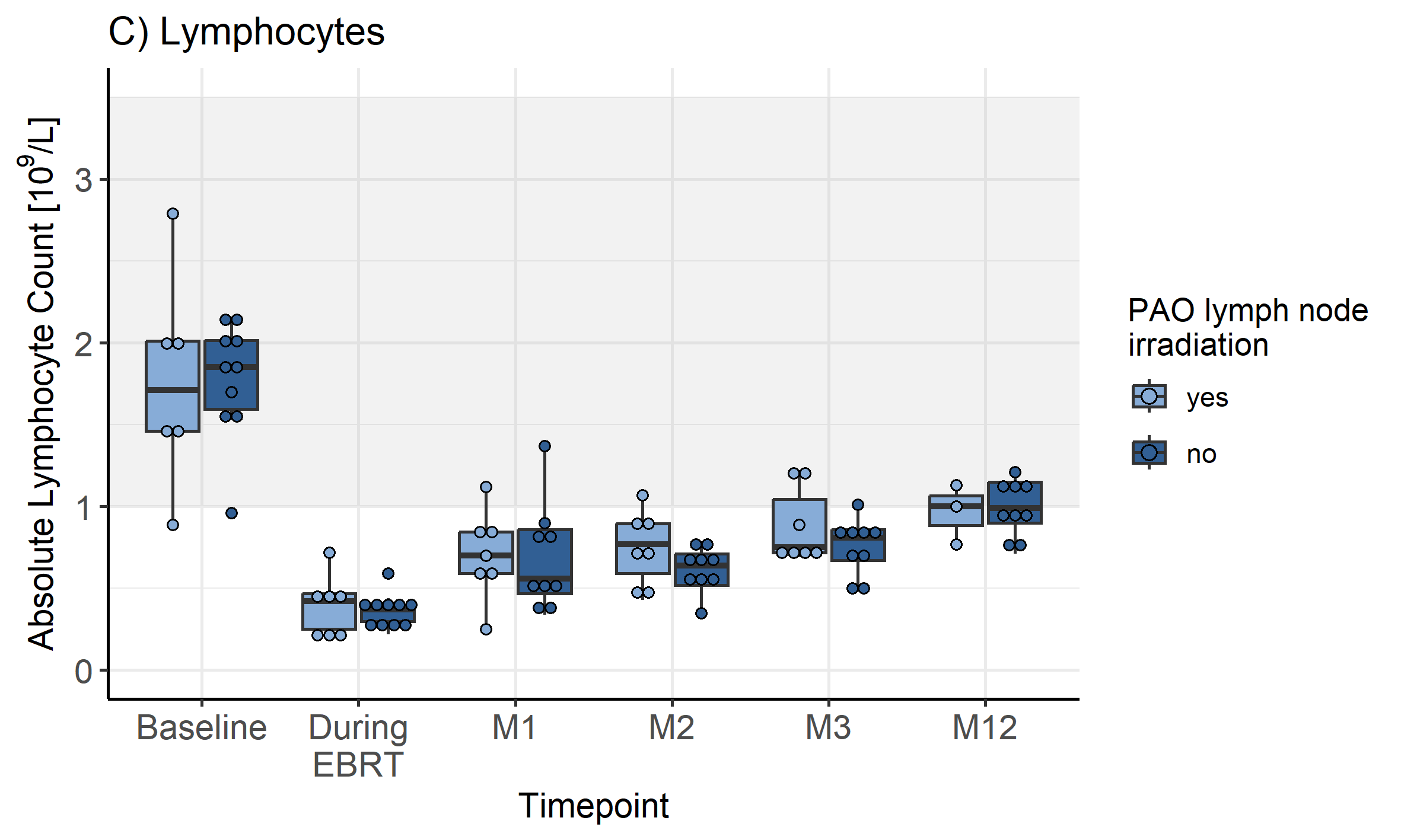

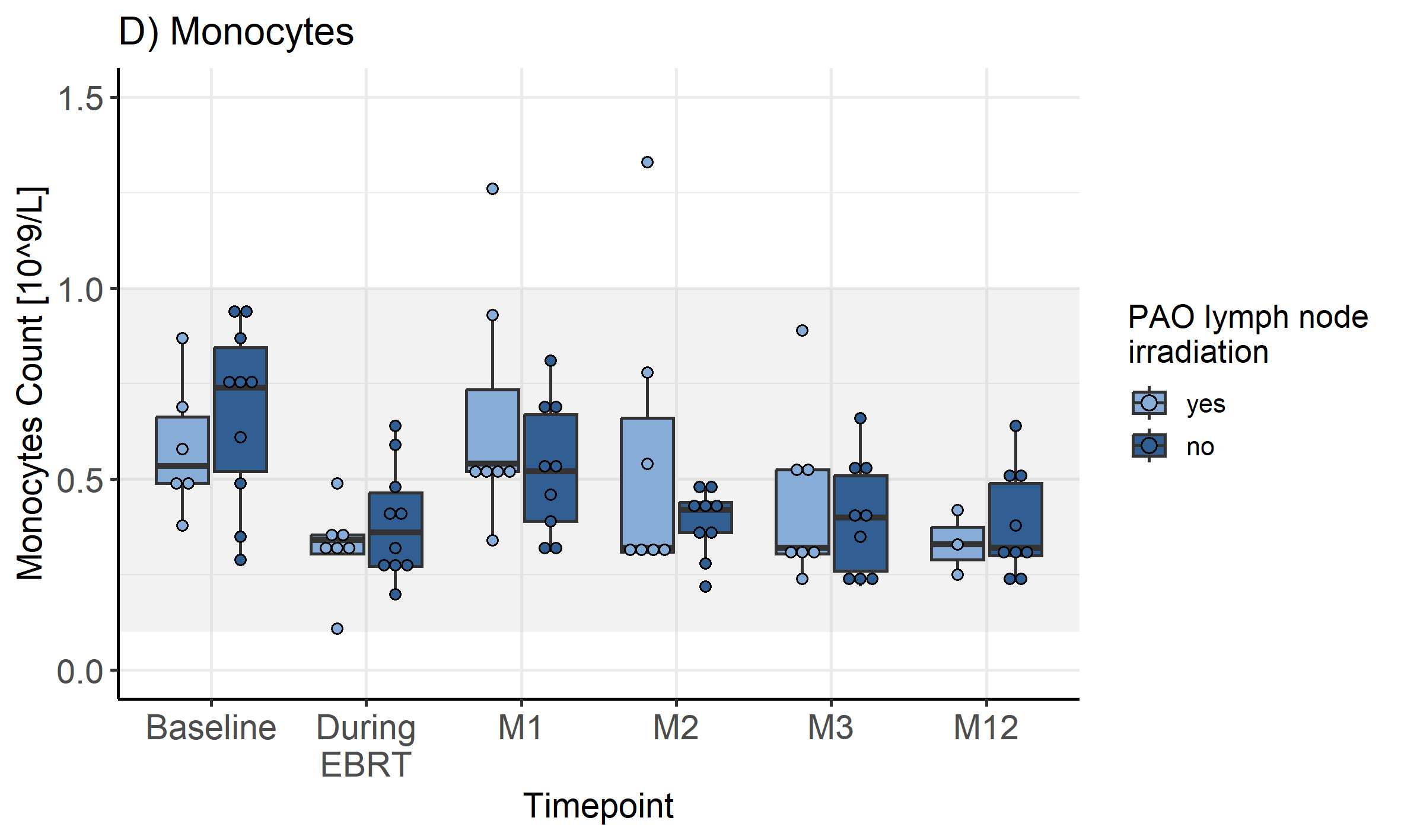
**

**Figure S1:** Complete blood cell counts of women with locally advanced cervical cancer (LACC) treated with bone marrow sparing volumetric modulated arc therapy (VMAT), subdivided into patients receiving or not receiving para-aortic (PAO) lymph node irradiation. Median fold changes relative to baseline are shown per timepoint. Boxes represent the interquartile range, whiskers indicate the full range. The grey area highlights the normal range according to the clinical practice. *EBRT = external beam radiation therapy, M = month*

**Table S3:** Linear mixed-effects models for fold changes in T-cell response to antigens A) influenza peptides (FLU) and B) memory response mix (MRM), tested with freshly isolated peripheral blood mononuclear cell (PBMCs) in a lymphocyte stimulation test (LST), and C) phytohemagglutinin (PHA), studied using cryopreserved PBMCs in a proliferation test, in women with locally advanced cervical cancer (LACC) treated with bone marrow sparing volumetric modulated arc therapy (VMAT). Median fold changes in response relative to baseline were compared for the acute (during treatment and up to 1 month) and late phase (6 weeks to 12 months post-treatment) and log10-transformed.

1. FLU

| Characteristic | Estimate | Standard Error | p-value |
| --- | --- | --- | --- |
| *Intercept* | 0.00 | 0.13 |  |
| *Timepoint* |  |  |  |
| acute | -0.65 | 0.14 | <0.001^*^ |
| late | -0.36 | 0.15 | 0.023^*^ |

*^*^ = statistically significant*

1. MRM

| Characteristic | Estimate | Standard Error | p-value |
| --- | --- | --- | --- |
| *Intercept* | 0.00 | 0.11 |  |
| *Timepoint* |  |  |  |
| acute | -0.32 | 0.11 | 0.004^*^ |
| late | -0.04 | 0.12 | 0.708 |

*^*^ = statistically significant*

1. PHA

| Characteristic | Estimate | Standard Error | p-value |
| --- | --- | --- | --- |
| *Intercept* | 0.00 | 0.15 |  |
| *Timepoint* |  |  |  |
| acute | 0.39 | 0.16 | 0.024^*^ |
| late | 0.17 | 0.17 | 0.323 |

*^*^ = statistically significant*

**Table S4:** Linear mixed-effects models for fold changes in antigen-presenting capacity, tested with mixed lymphocyte reaction (MLR), in women with locally advanced cervical cancer (LACC) treated with bone marrow sparing volumetric modulated arc therapy (VMAT). Fold changes were compared to baseline for the acute (during treatment and up to 1 month) and late phase (6 weeks to 12 months post-treatment) and log10-transformed.

| Characteristic | Estimate | Standard Error | p-value |
| --- | --- | --- | --- |
| *Intercept* | 0.00 | 0.04 |  |
| *Timepoint* |  |  |  |
| acute | -0.11 | 0.06 | 0.095 |
| late | 0.02 | 0.06 | 0.793 |

*^*^ = statistically significant*

**Table S5:** Linear mixed-effects models for fold changes in CD3, CD4+ T helper (CD3+CD4+ excluding regulatory T cells (CD3+CD4+CD25+CD127-Foxp3+)), and CD8+ (CD3+CD8+) T cells, based on flow cytometry, in women with locally advanced cervical cancer (LACC) treated with bone marrow sparing volumetric modulated arc therapy (VMAT). Fold changes were compared to baseline and log10-transformed.

1. CD3+ T cells

| Characteristic | Estimate | Standard Error | p-value |
| --- | --- | --- | --- |
| *Intercept* | 0.00 | 0.02 |  |
| *Timepoint* |  |  |  |
| During EBRT | -0.09 | 0.02 | <0.001^*^ |
| 1M after treatment | -0.13 | 0.02 | <0.001^*^ |
| 2M after treatment | -0.12 | 0.02 | <0.001^*^ |
| 3M after treatment | -0.12 | 0.02 | <0.001^*^ |
| 12M after treatment | -0.13 | 0.03 | <0.001^*^ |

*^*^ = statistically significant*

1. CD4+ T helper cells

| Characteristic | Estimate | Standard Error | p-value |
| --- | --- | --- | --- |
| *Intercept* | 0.00 | 0.02 |  |
| *Timepoint* |  |  |  |
| During EBRT | -0.09 | 0.03 | 0.001^*^ |
| 1M after treatment | -0.23 | 0.03 | <0.001^*^ |
| 2M after treatment | -0.21 | 0.03 | <0.001^*^ |
| 3M after treatment | -0.21 | 0.03 | <0.001^*^ |
| 12M after treatment | -0.20 | 0.03 | <0.001^*^ |

*^*^ = statistically significant*

1. CD8+ T cells

| Characteristic | Estimate | Standard Error | p-value |
| --- | --- | --- | --- |
| *Intercept* | 0.00 | 0.04 |  |
| *Timepoint* |  |  |  |
| During EBRT | -0.14 | 0.04 | <0.001^*^ |
| 1M after treatment | -0.01 | 0.04 | 0.758 |
| 2M after treatment | -0.01 | 0.04 | 0.892 |
| 3M after treatment | -0.01 | 0.04 | 0.860 |
| 12M after treatment | -0.06 | 0.04 | 0.188 |

*^*^ = statistically significant*

|  | **CD4+** | **CD8+** |
| --- | --- | --- |
| **Inhibitory markers** | 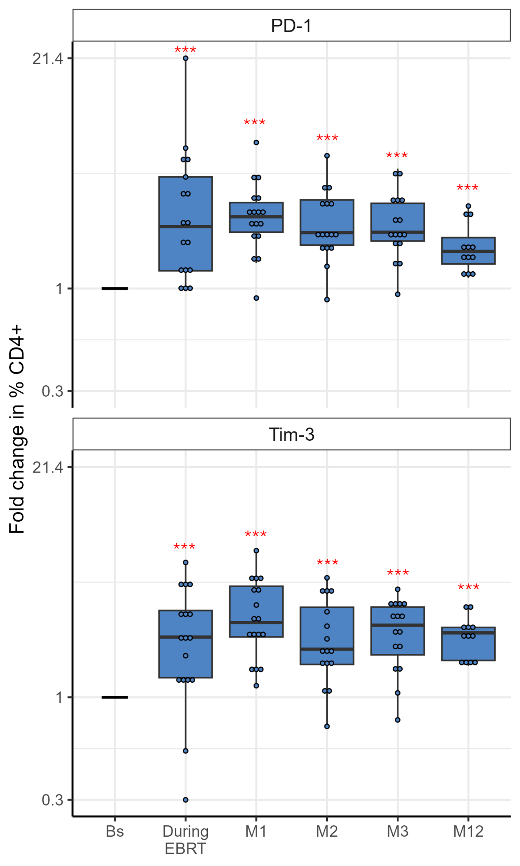  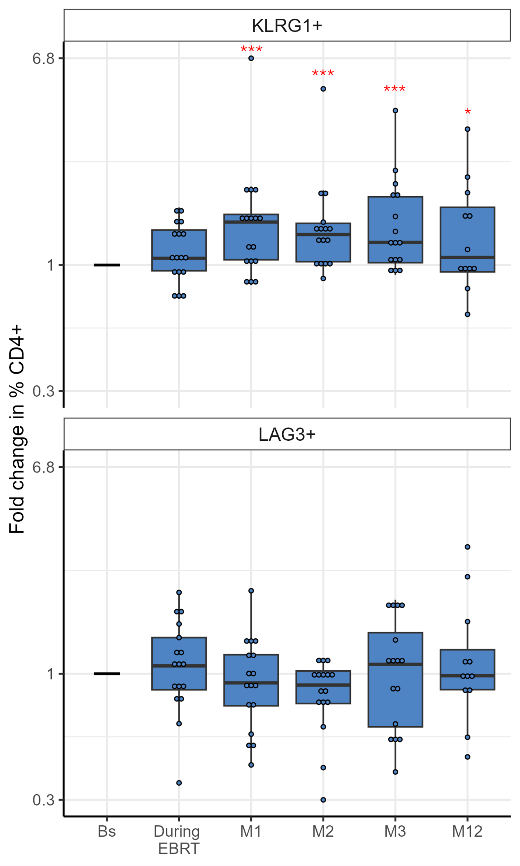 | 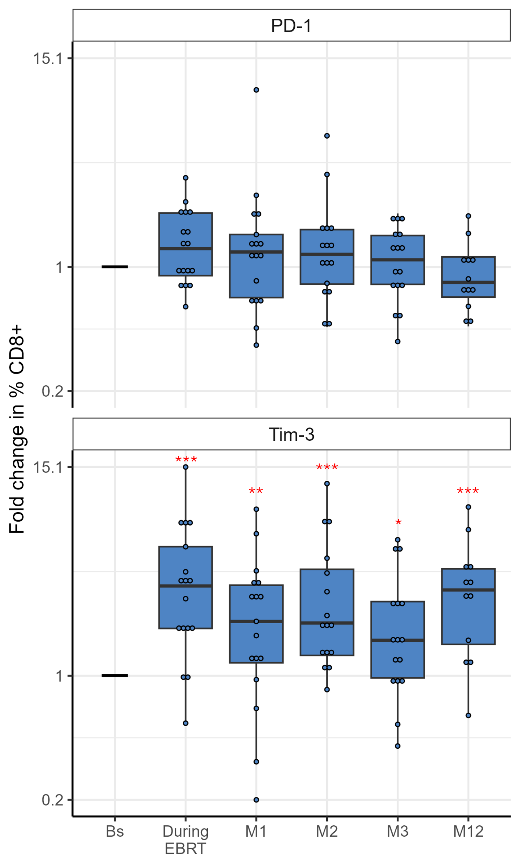  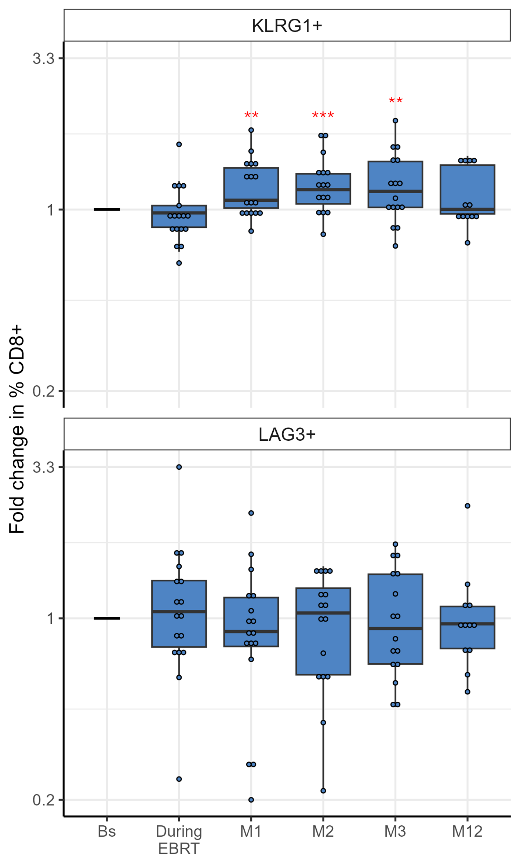 |

|  | **CD4+** | **CD8+** |
| --- | --- | --- |
| **Activation markers** | 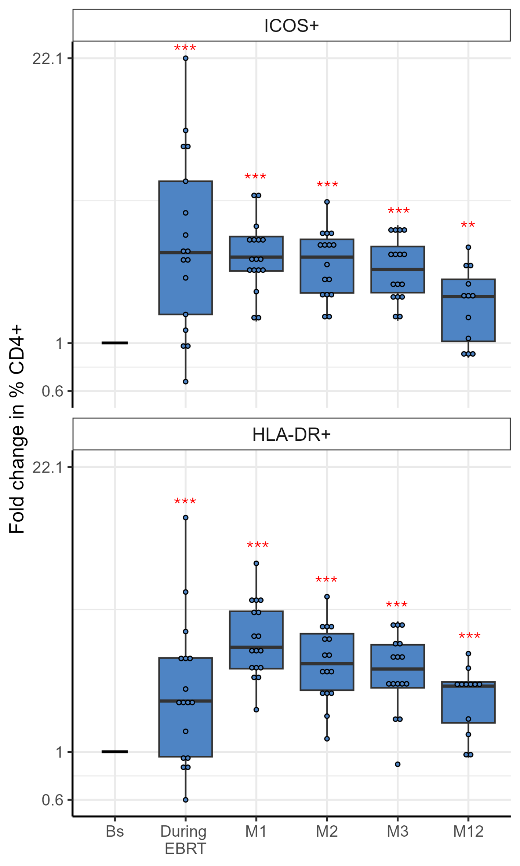 | 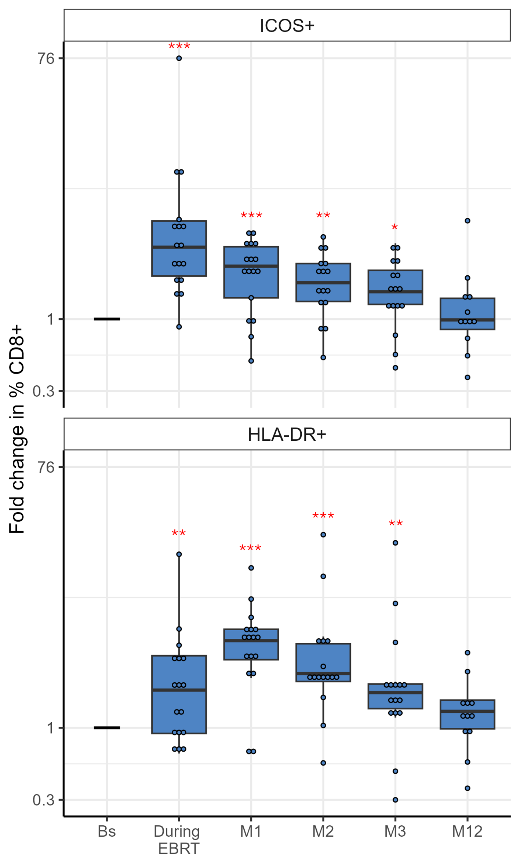 |
| **Tregs** | 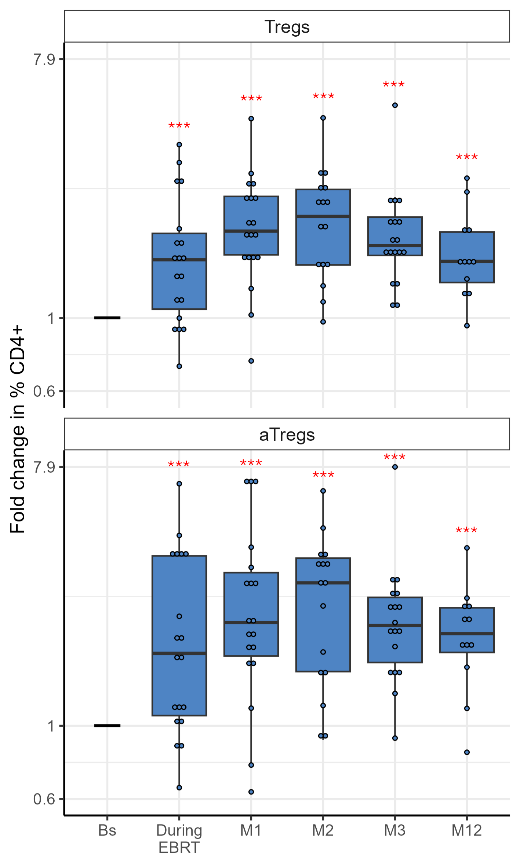 |  |

**Figure S2:** Fold changes over time for expression of inhibitory and activation markers, expressed as percentage of CD3+CD4+ or CD3+CD8+ T cells, as determined with flow cytometry in women with locally advanced cervical cancer (LACC) treated with bone marrow sparing volumetric-modulated arc therapy (VMAT). Median fold changes relative to baseline are shown per timepoint. Boxes represent the interquartile range, whiskers indicate the full range, and significance is indicated as *** = p<0.001, ** = p<0.01, * = p<0.05 (linear mixed-effects model).

**Table S6:** Linear mixed-effects models for fold changes in B cells (CD3-CD56-CD19+) and natural killer cells (CD3-CD56+CD19-), based on flow cytometry, in women with locally advanced cervical cancer (LACC) treated with bone marrow sparing volumetric modulated arc therapy (VMAT). Fold changes were compared to baseline and log10-transformed.

1. B cells

| Characteristic | Estimate | Standard Error | p-value |
| --- | --- | --- | --- |
| *Intercept* | 0.00 | 0.05 |  |
| *Timepoint* |  |  |  |
| During EBRT | -0.84 | 0.06 | <0.001^*^ |
| 1M after treatment | -0.59 | 0.06 | <0.001^*^ |
| 2M after treatment | -0.18 | 0.06 | 0.007^*^ |
| 3M after treatment | 0.02 | 0.06 | 0.788 |
| 12M after treatment | 0.23 | 0.07 | 0.002^*^ |

*^*^ = statistically significant*

1. Natural killer cells

| Characteristic | Estimate | Standard Error | p-value |
| --- | --- | --- | --- |
| *Intercept* | 0.00 | 0.04 |  |
| *Timepoint* |  |  |  |
| During EBRT | -0.07 | 0.04 | 0.079 |
| 1M after treatment | 0.04 | 0.04 | 0.275 |
| 2M after treatment | 0.08 | 0.04 | 0.054 |
| 3M after treatment | 0.11 | 0.04 | 0.011^*^ |
| 12M after treatment | 0.18 | 0.04 | <0.001^*^ |

*^*^ = statistically significant*

**Table S7:** Linear mixed-effects models for fold changes in myeloid cells (CD3-CD19-CD56-), myeloid-derived suppressor cells (MDSCs) (CD3-CD19-CD56-HLA-DR-), and myeloid-derived cells (CD3-CD19-CD56-HLA-DR+) and based on flow cytometry, in women with locally advanced cervical cancer (LACC) treated with bone marrow sparing volumetric modulated arc therapy (VMAT). Fold changes were compared to baseline and log10-transformed.

1. Myeloid cells

| Characteristic | Estimate | Standard Error | p-value |
| --- | --- | --- | --- |
| *Intercept* | 0.00 | 0.03 |  |
| *Timepoint* |  |  |  |
| During EBRT | 0.24 | 0.03 | <0.001^*^ |
| 1M after treatment | 0.22 | 0.03 | <0.001^*^ |
| 2M after treatment | 0.18 | 0.03 | <0.001^*^ |
| 3M after treatment | 0.16 | 0.03 | <0.001^*^ |
| 12M after treatment | 0.08 | 0.03 | 0.009^*^ |

*^*^ = statistically significant*

1. Myeloid-derived suppressor cells

| Characteristic | Estimate | Standard Error | p-value |
| --- | --- | --- | --- |
| *Intercept* | 0.00 | 0.03 |  |
| *Timepoint* |  |  |  |
| During EBRT | -0.14 | 0.03 | <0.001^*^ |
| 1M after treatment | -0.14 | 0.03 | <0.001^*^ |
| 2M after treatment | -0.11 | 0.03 | <0.001^*^ |
| 3M after treatment | -0.09 | 0.03 | 0.005^*^ |
| 12M after treatment | -0.06 | 0.03 | 0.073 |

*^*^ = statistically significant*

1. Myeloid-derived cells

| Characteristic | Estimate | Standard Error | p-value |
| --- | --- | --- | --- |
| *Intercept* | 0.00 | 0.01 |  |
| *Timepoint* |  |  |  |
| During EBRT | 0.03 | 0.01 | <0.001^*^ |
| 1M after treatment | 0.03 | 0.01 | <0.001^*^ |
| 2M after treatment | 0.02 | 0.01 | <0.001^*^ |
| 3M after treatment | 0.02 | 0.01 | <0.001^*^ |
| 12M after treatment | 0.02 | 0.01 | 0.005^*^ |

*^*^ = statistically significant*

**
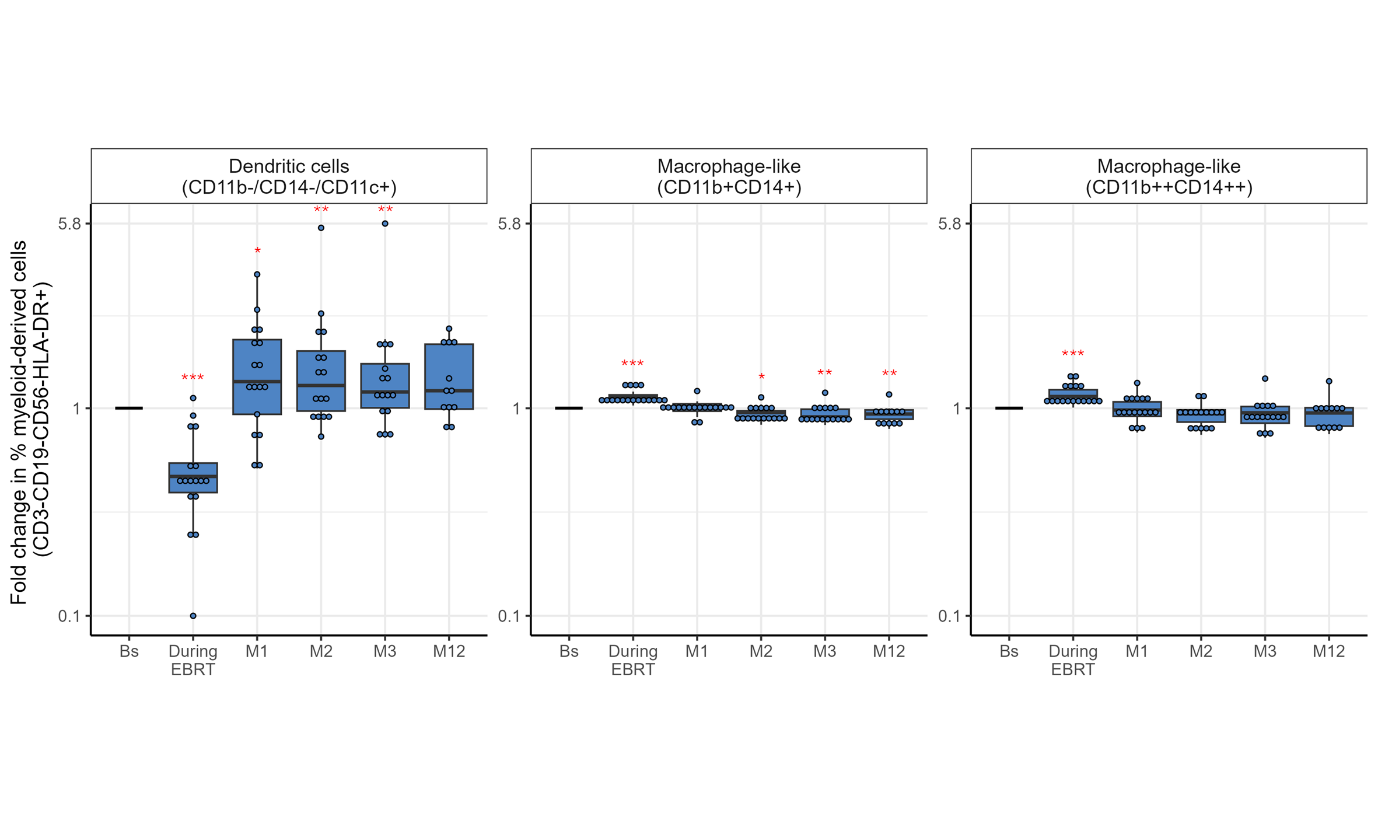
**

**Figure S3:** Fold changes over time for expression of subsets of myeloid-derived cells (CD3-CD19-CD56-HLA-DR+), including dendritic cells and macrophage-like cells, as percentage of myeloid-derived cells in women with locally advanced cervical cancer (LACC) treated with bone marrow sparing volumetric-modulated arc therapy (VMAT). Median fold changes relative to baseline are shown per timepoint. Boxes represent the interquartile range, whiskers indicate the full range, and significance is indicated as *** = p<0.001, ** = p<0.01, * = p<0.05 (linear mixed-effects model).

**Table S8**: Patient characteristics of women treated with bone marrow sparing (BMS) volumetric-modulated arc therapy (VMAT) and non-BMS three dimensional conformal radiation therapy (3DCRT) or intensity-modulated radiation therapy (IMRT). *EBRT = external beam radiation therapy, FIGO = International Federation of Gynecology and Obstetrics,* *PAO = para-aortic, sd = standard deviation*

| Characteristic | median/mean (range) or n [%] | |
| --- | --- | --- |
|  | **BMS VMAT**  **(n = 18)** | **non-BMS 3DCRT/IMRT**  **(n = 11)** |
| EBRT technique |  |  |
| VMAT | 18 [100%] | 0 |
| IMRT | 0 | 4 [36.4%] |
| 3DCRT | 0 | 7 [63.6%] |
| Median age [years] (range) | 52.5 (25 – 77) | 50 (28 – 70) |
| Smoking |  |  |
| yes | 2 [11.1%] | 1 [9.1%] |
| former | 4 [22.2%] | 3 [27.3%] |
| no | 12 [66.7%] | 6 [54.5%] |
| unknown | 0 | 1 [9.1%] |
| FIGO stage (2018) |  |  |
| IIB | 5 [27.8%] | 2 [18.2%] |
| IIIB | 1 [5.6%] | *NA* |
| IIIC1 | 12 [66.7%] | 7 [63.6%] |
| IIIC2 | *NA* | 2 [18.2%] |
| No. of chemotherapy cycles |  |  |
| 1 | 1 [5.6%] | 0 |
| 2 | 1 [5.6%] | 0 |
| 4 | 5 [27.8%] | 3 [27.3%] |
| 5 | 11 [61.1%] | 1 [9.1%] |
| 6 | *NA* | 7 [63.6%] |
| Pretreatment lymphocyte count |  |  |
| median [10^9^/L] (range) | 1.85 (0.89 – 2.79) | 1.80 (0.90 – 2.75) |
| missing | 2 [11.1%] | 1 [9.1%] |
| Pretreatment neutrophil/lymphocyte ratio |  |  |
| median (range) | 3.26 (1.35 – 8.16) | 2.74 (1.23 – 6.63) |
| missing | 2 [11.1%] | 1 [9.1%] |
| PAO lymph node irradiation |  |  |
| yes | 10 [55.6%] | 2 [18.2%] |
| no | 8 [44.4%] | 9 [81.8%] |
| Mean PTV volume [cm^3^] (sd) | 1,445.9 (259.6) | 1,963.6 (330.6) |
| Mean body V_10Gy_ [cm^3^] (sd) | 14,286 (3,627.4) | 14,223.8 (3,627.7) |
| Mean body V_36Gy_ [cm^3^] (sd) | 2,304.8 (525.9) | 4,099.5 (1,719.0) |
| Mean body V_43Gy_ [cm^3^] (sd) | 1,494.0 (307.6) | 3,540.5 (938.9) |
| Mean body V_50Gy_ [cm^3^] (sd) | 60.5 (59.6) | 933.4 (1,208.4) |
| Mean pelvic bones D_mean_ [Gy] (sd) | 24.7 (1.7) | 32.8 (19.8) |
| Mean volume pelvic bones [cm^3^] (sd) | 1,433.3 (174.1) | 1,539.4 (142.0) |
| Mean V_10Gy_ pelvic bones [%] (sd) | 86.1 (3.8) | 90.8 (3.6) |
| Mean V_20Gy_ pelvic bones [%] (sd) | 63.1 (4.3) | 83.2 (5.3) |
| Mean V_40Gy_ pelvic bones [%] (sd) | 13.3 (2.9) | 36.7 (5.9) |

**Table S9**: Linear mixed-effects models for complete blood cell counts in women with locally advanced cervical cancer (LACC). Women treated with chemoradiotherapy with women treated with volumetric-modulated arc therapy with bone marrow sparing (BMS VMAT) were exploratively compared with three dimensional conformal radiation therapy or intensity-modulated radiation therapy without bone marrow sparing (non-BMS 3DCRT/IMRT). *EBRT = external beam radiation therapy, M = month*

1. Leukocytes

| Characteristic | Estimate | Standard Error | p-value |
| --- | --- | --- | --- |
| *non-BMS 3DCRT/IMRT* |  |  |  |
| *Intercept* | 8.95 | 0.72 |  |
| *Timepoint* |  |  |  |
| During EBRT | -4.27 | 0.78 | <0.001^*^ |
| 1M after treatment | -4.29 | 0.87 | <0.001^*^ |
| 2M after treatment | -3.72 | 0.87 | <0.001^*^ |
| 3M after treatment | -3.90 | 0.87 | <0.001^*^ |
|  |  |  |  |
| *BMS VMAT [ref non-BMS 3DCRT/IMRT]* | |  |  |
| Baseline | -0.71 | 0.91 | 0.439 |
| During EBRT | -0.95 | 0.98 | 0.339 |
| 1M after treatment | 0.92 | 1.06 | 0.387 |
| 2M after treatment | -0.4 | 1.07 | 0.707 |
| 3M after treatment | -0.54 | 1.07 | 0.616 |

*^*^ = statistically significant*

1. Neutrophils

| Characteristic | Estimate | Standard Error | p-value |
| --- | --- | --- | --- |
| *non-BMS 3DCRT/IMRT* |  |  |  |
| *Intercept* | 5.03 | 0.59 |  |
| *Timepoint* |  |  |  |
| During EBRT | -1.81 | 0.69 | 0.011^*^ |
| 1M after treatment | -2.30 | 0.74 | 0.002^*^ |
| 2M after treatment | -1.67 | 0.74 | 0.025^*^ |
| 3M after treatment | -1.69 | 0.74 | 0.024^*^ |
|  |  |  |  |
| *BMS VMAT [ref non-BMS 3DCRT/IMRT]* | |  |  |
| Baseline | 0.49 | 0.75 | 0.521 |
| During EBRT | -0.52 | 0.84 | 0.538 |
| 1M after treatment | 1.14 | 0.88 | 0.200 |
| 2M after treatment | 0.02 | 0.88 | 0.980 |
| 3M after treatment | -0.22 | 0.88 | 0.804 |

*^*^ = statistically significant*

1. Lymphocytes

| Characteristic | Estimate | Standard Error | p-value |
| --- | --- | --- | --- |
| *non-BMS 3DCRT/IMRT* |  |  |  |
| *Intercept* | 1.82 | 0.10 |  |
| *Timepoint* |  |  |  |
| During EBRT | -1.40 | 0.11 | <0.001^*^ |
| 1M after treatment | -1.18 | 0.12 | <0.001^*^ |
| 2M after treatment | -1.08 | 0.11 | <0.001^*^ |
| 3M after treatment | -1.21 | 0.11 | <0.001^*^ |
|  |  |  |  |
| *BMS VMAT [ref non-BMS 3DCRT/IMRT]* | |  |  |
| Baseline | -0.04 | 0.12 | 0.736 |
| During EBRT | -0.04 | 0.14 | 0.792 |
| 1M after treatment | 0.05 | 0.14 | 0.738 |
| 2M after treatment | -0.06 | 0.14 | 0.687 |
| 3M after treatment | 0.18 | 0.14 | 0.197 |

*^*^ = statistically significant*

1. Monocytes

| Characteristic | Estimate | Standard Error | p-value |
| --- | --- | --- | --- |
| *non-BMS 3DCRT/IMRT* |  |  |  |
| *Intercept* | 0.70 | 0.06 |  |
| *Timepoint* |  |  |  |
| During EBRT | -0.39 | 0.07 | <0.001^*^ |
| 1M after treatment | -0.32 | 0.08 | <0.001^*^ |
| 2M after treatment | -0.28 | 0.07 | <0.001^*^ |
| 3M after treatment | -0.33 | 0.07 | <0.001^*^ |
|  |  |  |  |
| *BMS VMAT [ref non-BMS 3DCRT/IMRT]* | |  |  |
| Baseline | -0.06 | 0.08 | 0.443 |
| During EBRT | 0.08 | 0.09 | 0.407 |
| 1M after treatment | 0.19 | 0.10 | 0.045^*^ |
| 2M after treatment | 0.04 | 0.09 | 0.678 |
| 3M after treatment | 0.05 | 0.09 | 0.571 |

*^*^ = statistically significant*

**Table S10:** Linear mixed-effects models for fold changes in T-cell response to antigens A) influenza peptides (FLU) and B) memory response mix (MRM), tested with freshly isolated peripheral blood mononuclear cell (PBMCs) in a lymphocyte stimulation test (LST), in women with locally advanced cervical cancer (LACC). Women treated with chemoradiotherapy with three dimensional conformal radiation therapy or intensity-modulated radiation therapy without bone marrow sparing (BMS) (non-BMS 3DCRT/IMRT) were exploratively compared to women treated with volumetric-modulated arc therapy with bone marrow sparing (BMS VMAT). Fold changes were compared to baseline for the acute (during treatment and up to 1 month) and late phase (6 weeks to 12 months post-treatment) and log10-transformed.

1. FLU

| Characteristic | Estimate | Standard Error | p-value |
| --- | --- | --- | --- |
| *Intercept* | 0 | 0.13 |  |
| *non-BMS 3DCRT/IMRT* |  |  |  |
| *Timepoint* |  |  |  |
| acute | -0.29 | 0.16 | 0.076 |
| late | -0.11 | 0.19 | 0.546 |
| *BMS VMAT [ref non-BMS 3DCRT/IMRT]* | | | |
| *Timepoint* |  |  |  |
| acute | -0.37 | 0.18 | 0.045^*^ |
| late | -0.25 | 0.22 | 0.261 |

*^*^ = statistically significant*

1. MRM

| Characteristic | Estimate | Standard Error | p-value |
| --- | --- | --- | --- |
| *Intercept* | 0 | 0.11 |  |
| *non-BMS 3DCRT/IMRT* |  |  |  |
| *Timepoint* |  |  |  |
| acute | -0.27 | 0.12 | 0.036^*^ |
| late | -0.13 | 0.14 | 0.387 |
| *BMS VMAT [ref non-BMS 3DCRT/IMRT]* | |  |  |
| *Timepoint* |  |  |  |
| acute | -0.05 | 0.13 | 0.678 |
| late | 0.09 | 0.16 | 0.594 |

*^*^ = statistically significant*


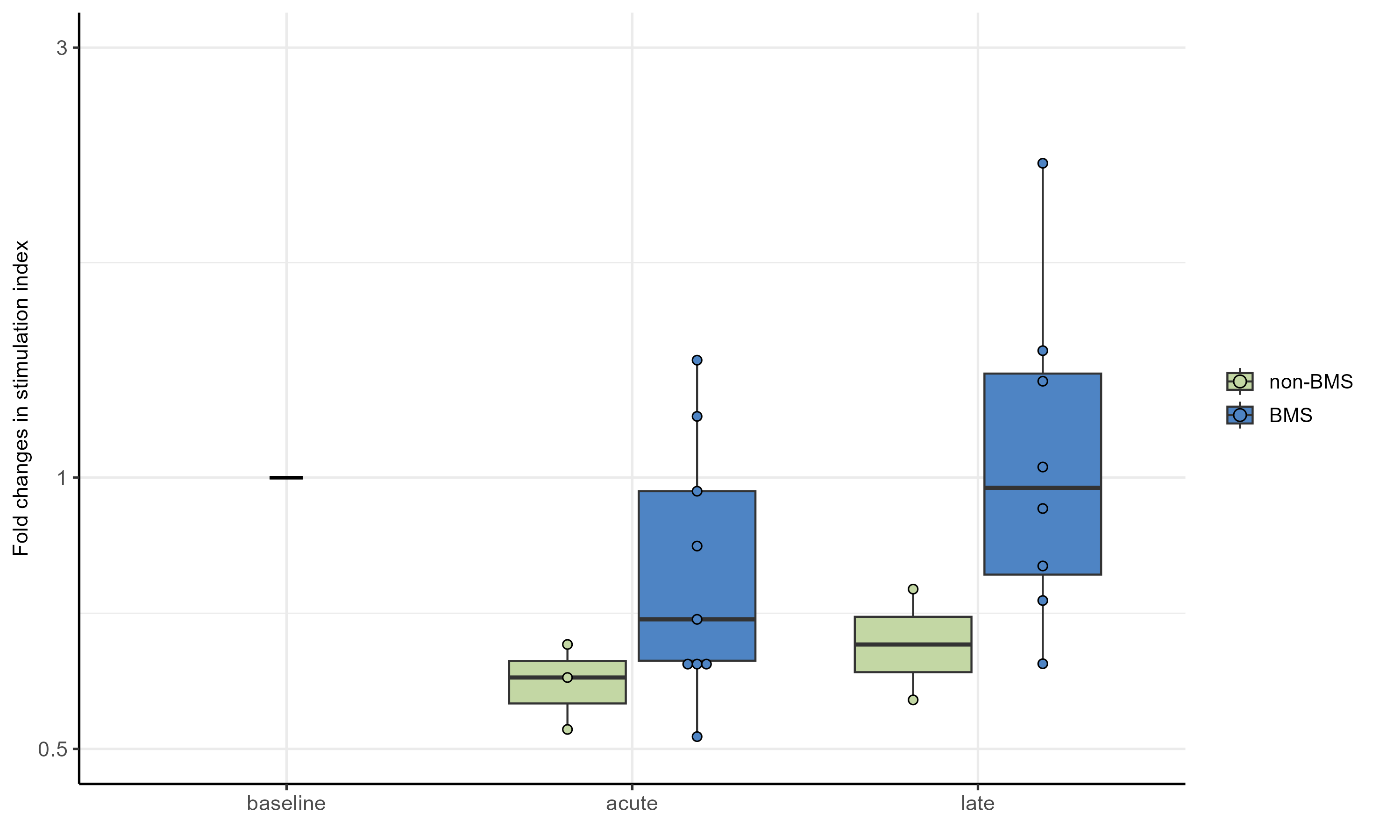


**Figure S4:** Fold changes in antigen-presenting capacity of peripheral blood mononuclear cell (PBMCs) as determined in a mixed lymphocyte reaction (MLR) in women with locally advanced cervical cancer (LACC) treated with chemoradiotherapy with three dimensional conformal radiation therapy (3DCRT) or intensity-modulated radiation therapy (IMRT) without bone marrow sparing (BMS) (non-BMS group) or with volumetric-modulated arc therapy (VMAT) with bone marrow sparing (BMS group). Median fold changes relative to baseline are shown per timepoint. Boxes represent the interquartile range, whiskers indicate the full range. None of the fold changes was statistically significant in the BMS group and this was not statistically tested in the non-BMS group as there were only three patients available for analysis.


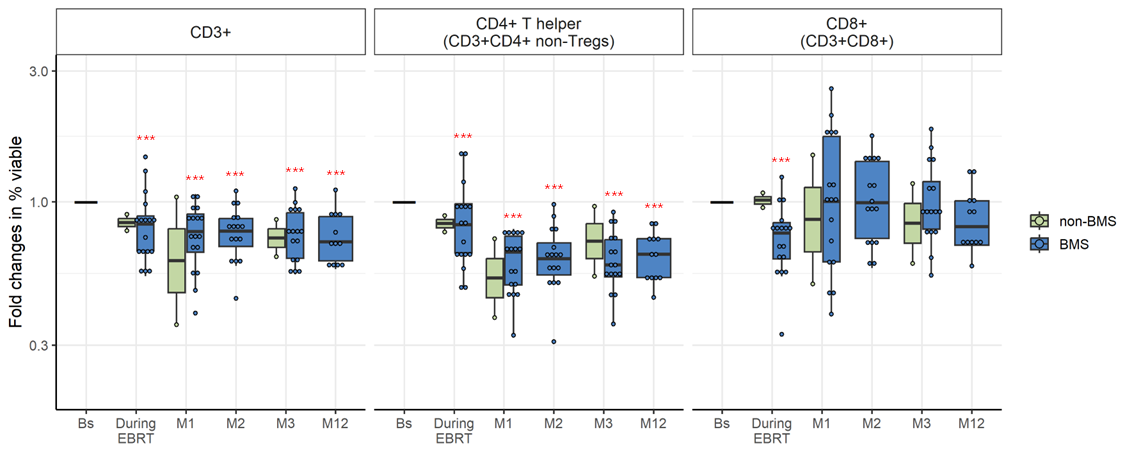


**Figure S5:** Fold changes in CD3+, CD4+ T helper (CD3+CD4+ excluding regulatory T cells (CD3+CD4+CD25+CD127-Foxp3+)), and CD8+ (CD3+CD8+) cell frequency, expressed as percentage of all viable cells, as determined with flow cytometry in women with locally advanced cervical cancer (LACC) treated with chemoradiotherapy with three dimensional conformal radiation therapy (3DCRT) or intensity-modulated radiation therapy (IMRT) without bone marrow sparing (BMS) (non-BMS group) or with volumetric-modulated arc therapy (VMAT) with bone marrow sparing (BMS group). Median fold changes relative to baseline are shown per timepoint. Boxes represent the interquartile range, whiskers indicate the full range, and significance is indicated as *** = p<0.001, ** = p<0.01, * = p<0.05 (linear mixed-effects model). This was not statistically tested in the non-BMS group as there were only three patients available for analysis.

|  | **CD4+** | **CD8+** |
| --- | --- | --- |
| **Inhibitory markers** | 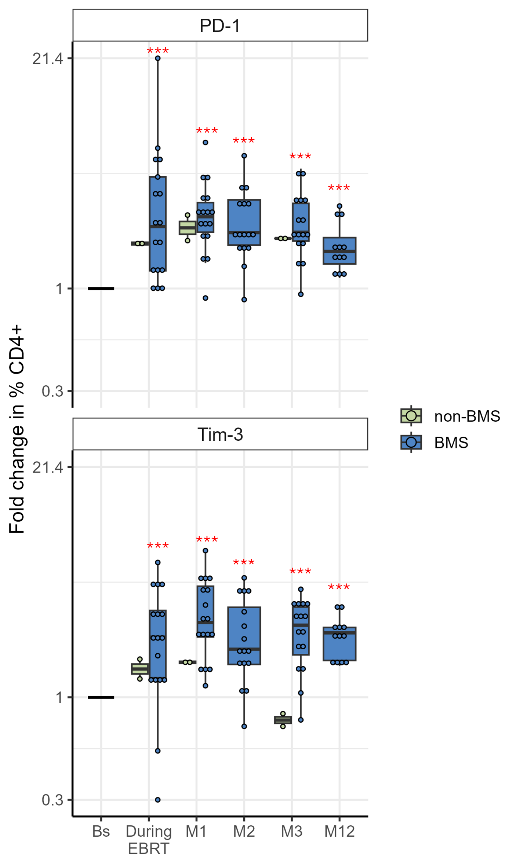 | 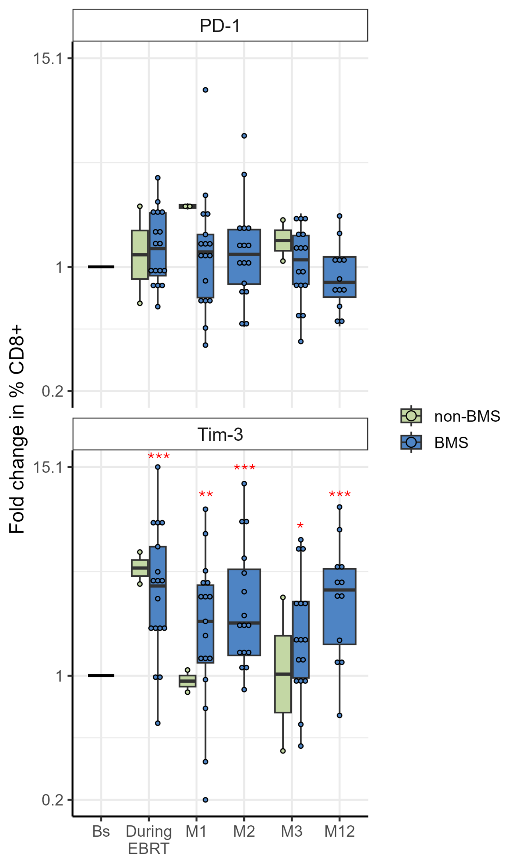 |
| **Tregs** | 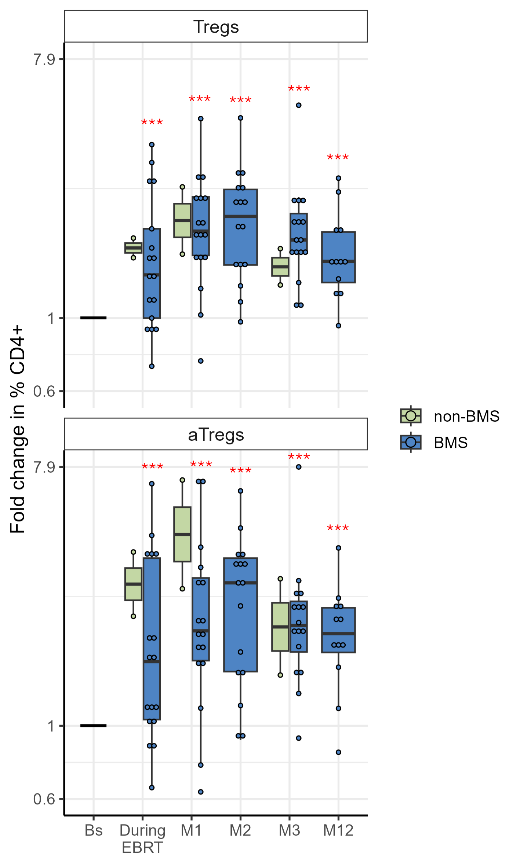 |  |

**Figure S6:** Fold changes over time for expression of inhibitory and activation markers as percentage of CD3+CD4+ or CD3+CD8+ T cells in women with locally advanced cervical cancer (LACC) treated with chemoradiotherapy with three dimensional conformal radiation therapy (3DCRT) or intensity-modulated radiation therapy (IMRT) without bone marrow sparing (BMS) (non-BMS group) or with volumetric-modulated arc therapy (VMAT) with bone marrow sparing (BMS group). Median fold changes relative to baseline are shown per timepoint. Boxes represent the interquartile range, whiskers indicate the full range, and significance is indicated as *** = p<0.001, ** = p<0.01, * = p<0.05 (linear mixed-effects model). This was not statistically tested in the non-BMS group as there were only three patients available for analysis.


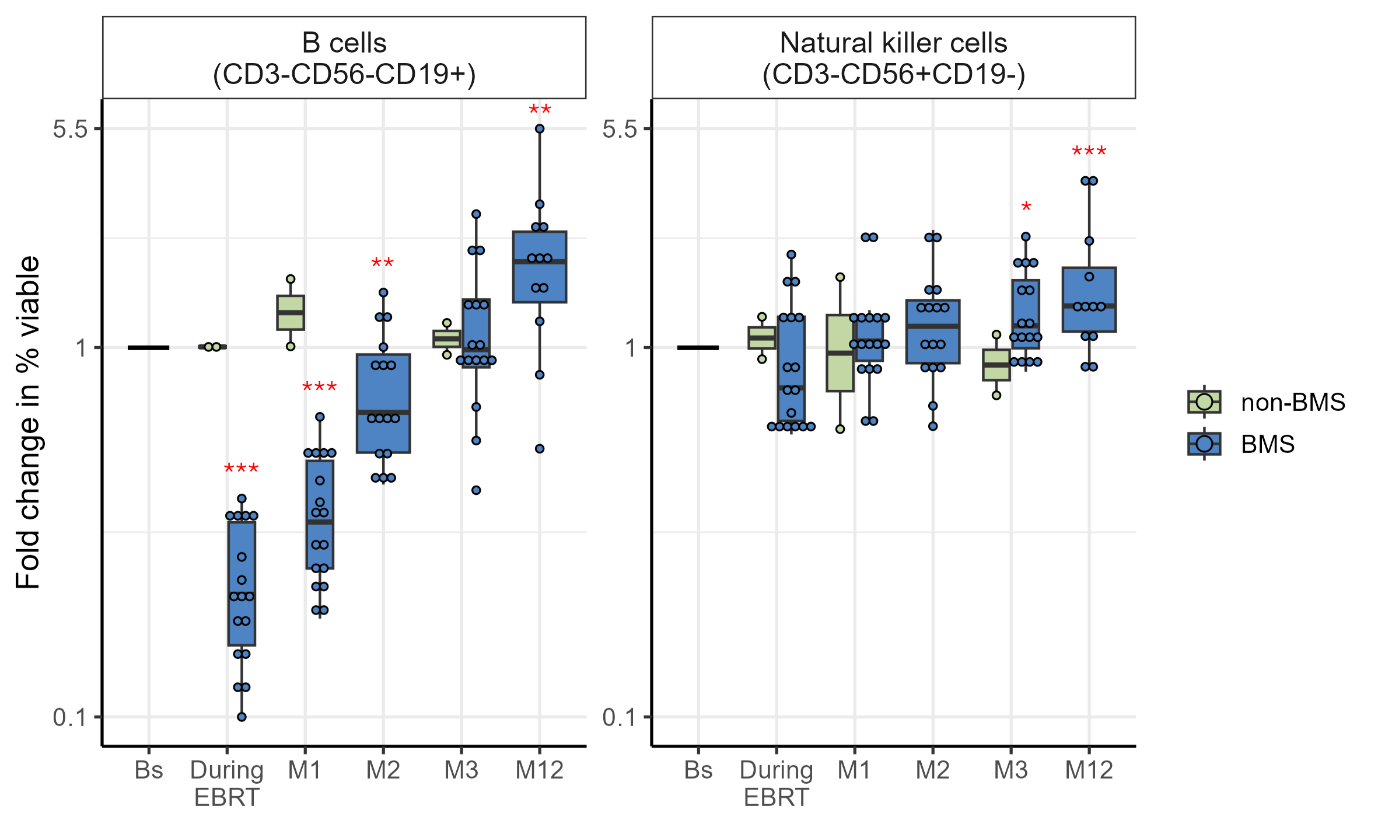


**Figure S7:** Fold changes over time for expression of B cells and natural killer (NK) cells, expressed as percentage of viable cells, in women with locally advanced cervical cancer (LACC) treated with chemoradiotherapy with three dimensional conformal radiation therapy (3DCRT) or intensity-modulated radiation therapy (IMRT) without bone marrow sparing (BMS) (non-BMS group) or with volumetric-modulated arc therapy (VMAT) with bone marrow sparing (BMS group). Median fold changes relative to baseline are shown per timepoint. Boxes represent the interquartile range, whiskers indicate the full range, and significance is indicated as *** = p<0.001, ** = p<0.01, * = p<0.05 (linear mixed-effects model). This was not statistically tested in the non-BMS group as there were only three patients available for analysis.


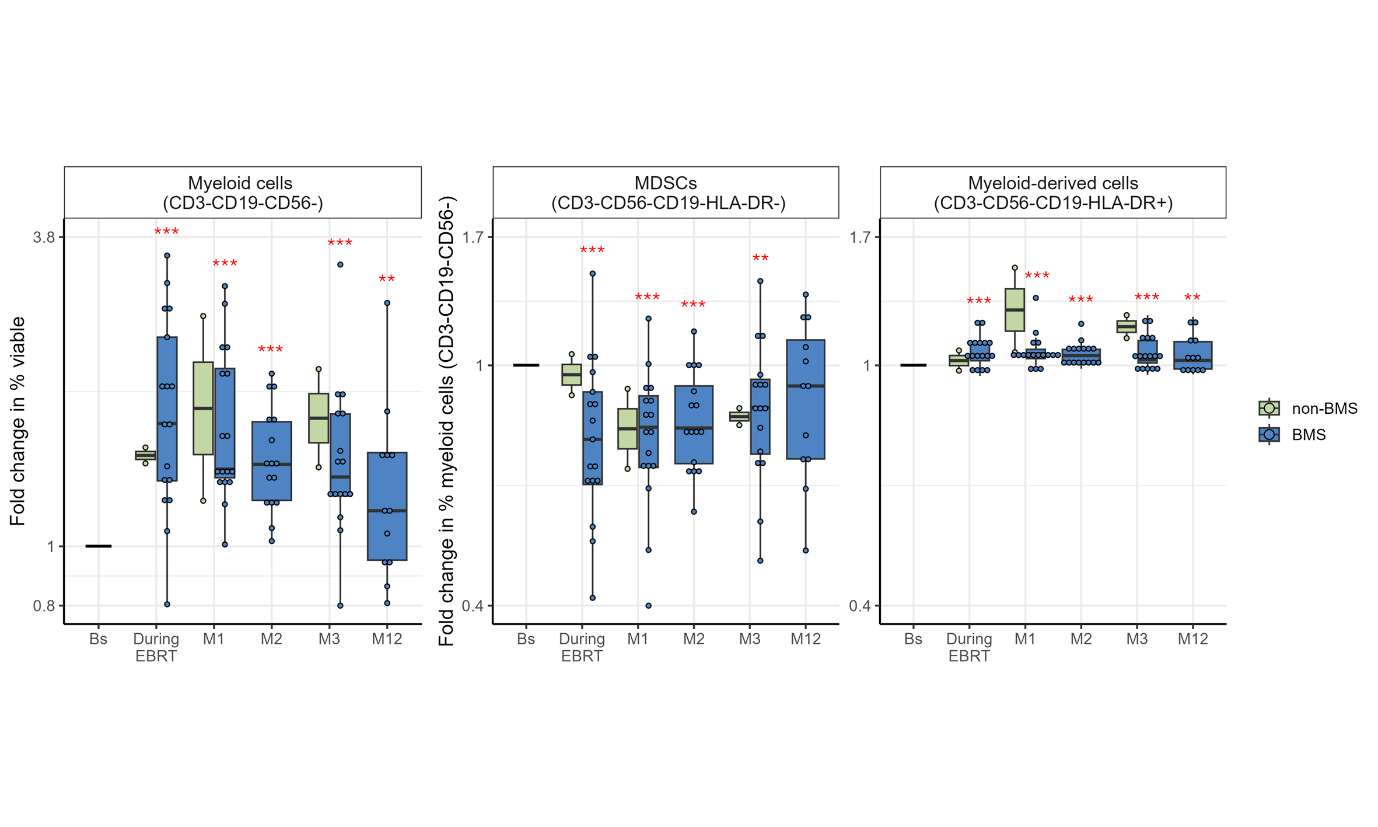


**Figure S8:** Fold changes over time for expression of myeloid cells (CD3-CD19-CD56-), as percentage of viable or CD45+ cells, and within this group the expression of myeloid-derived suppressor cells (MDSCs) (HLA-DR-) and myeloid-derived cells (HLA-DR+) in women with locally advanced cervical cancer (LACC) treated with chemoradiotherapy with three dimensional conformal radiation therapy (3DCRT) or intensity-modulated radiation therapy (IMRT) without bone marrow sparing (BMS) (non-BMS group) or with volumetric-modulated arc therapy (VMAT) with bone marrow sparing (BMS group). Median fold changes relative to baseline are shown per timepoint. Boxes represent the interquartile range, whiskers indicate the full range, and significance is indicated as *** = p<0.001, ** = p<0.01, * = p<0.05 (linear mixed-effects model). This was not statistically tested in the non-BMS group as there were only three patients available for analysis.


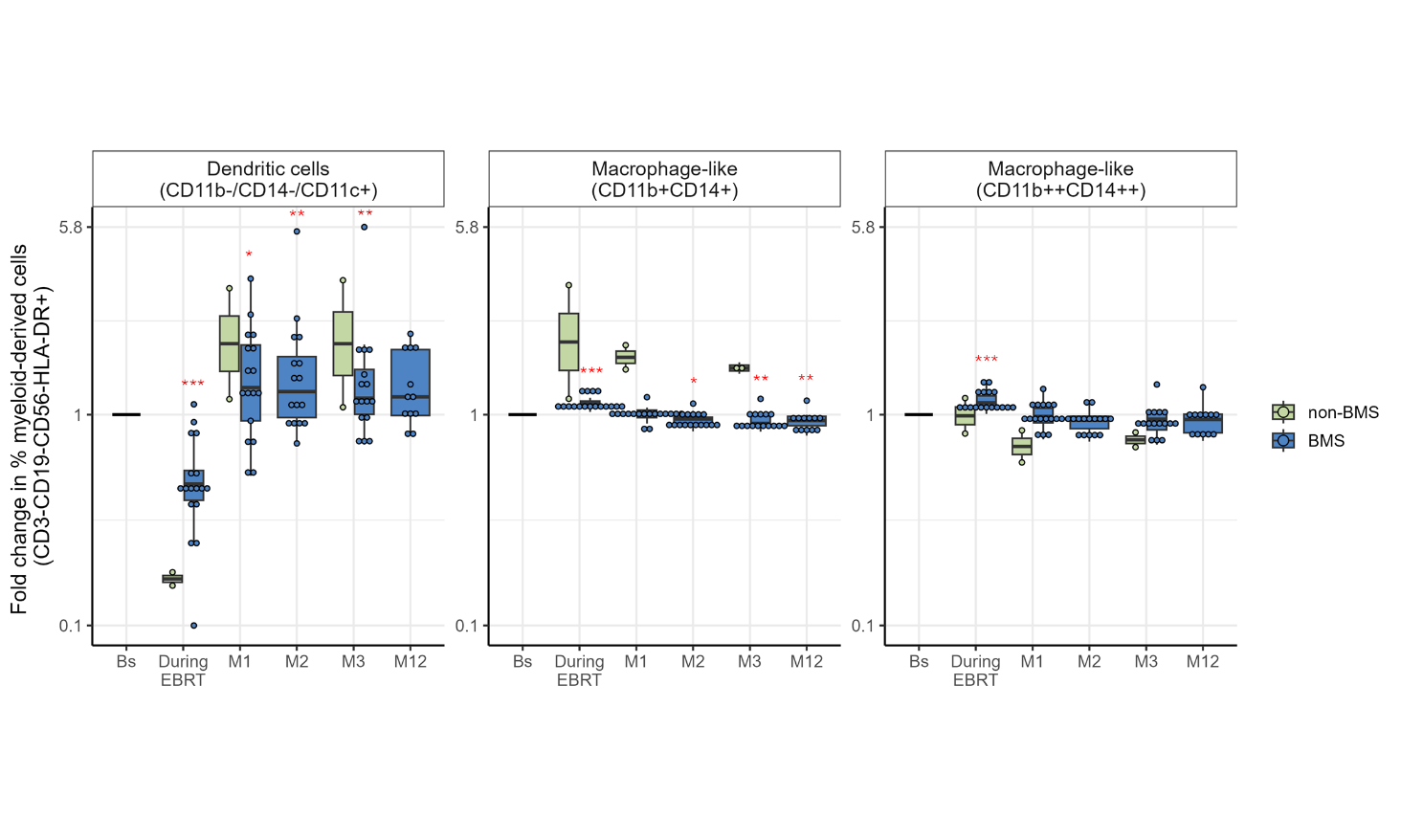


**Figure S9:** Fold changes over time for expression of subsets of myeloid-derived cells (CD3-CD19-CD56-HLA-DR+), including dendritic cells and macrophage-like cells, as percentage of myeloid-derived cells +) in women with locally advanced cervical cancer (LACC) treated with chemoradiotherapy with three dimensional conformal radiation therapy (3DCRT) or intensity-modulated radiation therapy (IMRT) without bone marrow sparing (BMS) (non-BMS group) or with volumetric-modulated arc therapy (VMAT) with bone marrow sparing (BMS group). Median fold changes relative to baseline are shown per timepoint. Boxes represent the interquartile range, whiskers indicate the full range, and significance is indicated as *** = p<0.001, ** = p<0.01, * = p<0.05 (linear mixed-effects model). This was not statistically tested in the non-BMS group as there were only three patients available for analysis.
